# Altered maturation and activation state of circulating monocytes is associated with their enhanced recruitment in pulmonary arterial hypertension

**DOI:** 10.1186/s12931-025-03182-0

**Published:** 2025-04-15

**Authors:** Rebecca L. Harper, Xin Zhou, David P. Marciano, Aiqin Cao, Lingli Wang, Guibin Chen, Mir S. Adil, Wenyu Zhou, Peter Maguire, Shanthi Deivanayagam, Quan Yu, Vignesh Viswanathan, Dan Yang, Marcy Martin, Sarasa Isobe, Shoichiro Otsuki, Jordan Burgess, Audrey Inglis, Devon Kelley, Patricia A. del Rosario, Andrew Hsi, Francois Haddad, Roham T. Zamanian, Manfred Boehm, Michael P. Snyder, Marlene Rabinovitch

**Affiliations:** 1 Department of Pediatrics, Stanford University School of Medicine, CCSR-1215A, 269 Campus Drive, Stanford, CA 94305-5162 USA; 2 Basic Science and Engineering (BASE) Initiative, Betty Irene Moore Children’s Heart Center, Lucile Packard Children’s Hospital, Stanford, CA 94305 USA; 3 Stanford Cardiovascular Institute, Stanford University School of Medicine, Stanford, CA 94305 USA; 4 Department of Genetics, Stanford University School of Medicine, Stanford, CA 94305 USA; 5 National Heart, Lung & Blood Institute, National Institutes of Health, Bethesda, MD 20800 USA; 6 Department of Radiation Oncology, Stanford University School of Medicine, Stanford, CA 94305 USA; 7 Department of Medicine, Stanford University School of Medicine, Stanford, CA 94305 USA; 8 Vera Moulton Wall Center for Pulmonary Vascular Diseases, Stanford University School of Medicine, Stanford, CA 94305 USA

**Keywords:** Monocyte, Pulmonary arterial hypertension, Macrophage, STAT1, BMPR2, CD14

## Abstract

**Background:**

It is well-established that patients with pulmonary arterial hypertension (PAH) exhibit increased recruitment of circulating monocytes to their pulmonary arteries. However, it remains unclear whether these monocytes have intrinsic abnormalities that contribute to their recruitment and to PAH pathogenesis. This study aimed to characterize the gene expression profiles of circulating classical, intermediate, and non-classical monocytes and assess their maturation trajectory in patients with idiopathic (I) PAH compared to control subjects. Additionally, it sought to explore the relationship between the observed IPAH abnormalities and deficiencies in bone morphogenetic receptor 2 (BMPR2), the most frequently mutated gene in PAH, and to assess adhesion and transendothelial migration, key processes in monocyte infiltration of pulmonary arteries.

**Methods:**

Differentially expressed genes and maturation trajectories of circulating monocytes from patients with IPAH vs. control subjects were compared using single cell RNA sequencing (scRNAseq), followed by FACS analysis. Observations from IPAH and control cells were related to reduced *BMPR2* using a THP1 monocyte cell line with *BMPR2* reduced by siRNA as well as induced pluripotent stem cell (iPSC) derived monocytes (iMono) from hereditary (H) PAH patients with a *BMPR2* mutation and monocytes from mice with *Bmpr2* deleted (MON-*Bmpr2*^*−/−*^*)*.

**Results:**

Classical IPAH monocytes have decreased *CD14* mRNA leading to a deviation in their maturation trajectory and early terminal fate, which is not rescued by cytokine treatment. Monocytes that evade early cell death show elevated *STAT1*, *PPDPF* and *HLA-B*, and an interferon (IFN) signature indicative of an altered activation state. A strong link between decreased *BMPR2* and *CD14* was observed in THP1 cells and in HPAH iMono with a *BMPR2* mutation associated with *STAT1* and *IFN* related genes, and in monocytes from MON-*Bmpr2*^−/−^ mice. Increased adhesion to iPSC-derived endothelial cells (iECs) in HPAH-*BMPR2* mutant iMono was associated with elevated ICAM1 expression. Enhanced transendothelial migration of these cells was associated with the reduction in endothelial VE-cadherin (CDH5).

**Conclusions:**

IPAH monocytes exhibit an altered activation state associated with reduced *BMPR2* and *CD14*, along with elevated *STAT1-IFN* expression. These changes are linked to intrinsic functional abnormalities that contribute to the monocytes’ increased propensity to invade the pulmonary circulation.

**Supplementary Information:**

The online version contains supplementary material available at 10.1186/s12931-025-03182-0.

## Background

Pulmonary arterial hypertension (PAH) is characterized by a progressive increase in the mean pulmonary arterial pressure leading to right-sided heart failure as distal pulmonary arteries become occluded due to vascular dysfunction and chronic inflammation [1, 2]. Vascular dysfunction can result from reduced expression of bone morphogenetic protein receptor type 2 (BMPR2). While *BMPR2* mutations are observed in hereditary PAH (HPAH) patients, reduced BMPR2 function is also evident in idiopathic (I) PAH without a mutation, and in PAH resulting from a number of primary medical conditions [3]. In pulmonary arterial endothelial cells (PAEC), reduced BMPR2 can lead to increased recruitment of inflammatory cells particularly myeloid cells [4], but the contribution of reduced BMPR2 to the intrinsic dysfunction of these cells has not been extensively investigated. Recent studies from our group using multiplex ion-based imaging revealed more severe myeloid cell inflammation in the lesions of patients with a *BMPR2* or *SMAD9* mutation [5].

The role of macrophages in the pathogenesis of PAH has been investigated in both clinical and experimental settings [1, 6]. Macrophages accumulate in the perivascular space where they drive chronic inflammation that contributes to obliteration of distal pulmonary arteries by inducing endothelial cell dysfunction as well as fibroblast and smooth muscle cell proliferation [1, 7–9]. PAH-associated macrophages exhibit a pro-inflammatory and anti-phagocytic phenotype, secreting cytokines and chemokines that shape the local tissue microenvironment. Importantly, experimental pulmonary hypertension (PH) can be prevented or reversed by depleting macrophages or the factors they release [4, 6].

Far fewer studies have examined monocytes in PAH. However, in two animal models of PH, vascular remodeling was attenuated by blocking recruitment of circulating monocytes from the bone marrow [6, 9, 10]. It is therefore becoming evident that the pathological role of macrophages in PAH results from recruitment and differentiation of circulating monocytes, rather than resident macrophages [9, 11]. Investigating the intrinsic properties of circulating monocytes is therefore important in understanding their role and that of their derivatives in PAH pathogenesis [12].

Classical monocytes (CD14^+^/CD16^−^) are recruited from the bone marrow to the systemic circulation, where they normally differentiate into intermediate (CD14^+^/CD16^+^) monocytes, which subsequently mature into non-classical (CD14^−^/CD16^+^) monocytes. Monocytes that fail to complete this differentiation process undergo apoptosis [13]. Normal CD14^+^ monocytes can differentiate into pro-inflammatory macrophages, while CD16 + monocytes typically give rise to anti-inflammatory macrophages [12, 14, 15]. Additionally, monocytes can also differentiate into monocyte derived dendritic cells that we have related to proliferating SMC [5].

In this study, we used single cell RNA sequencing (scRNAseq) to analyze the transcriptome of circulating monocytes from patients with IPAH compared to healthy controls. Trajectory analysis revealed two distinct transcriptomic profiles in IPAH monocytes. One population follows the typical phenotypic pathway from classical, to intermediate into non-classical while maintaining increased signal transducer and activator of transcription 1 (*STAT1*) gene expression. The second population, which fails to mature beyond the classical phenotype, exhibits low expression of *CD14*, and proto-oncogenes *FOS* and *JUNB.* Further investigation of this second classical monocyte population revealed a propensity towards cell death consistent with reduced *CD14* expression, and impaired response to activating stimuli.

We identified a link between reduced *CD14* and decreased BMPR2 levels in human monocytes, with a corresponding a decrease in the paralogue Ly6c in murine monocytes lacking *Bmpr2*. In response to hypoxia, *Bmpr2-d*eficient murine monocytes appear responsible for the increase in perivascular macrophages, consistent with enhanced monocyte recruitment and differentiation within the lung tissue. Human induced pluripotent stem cells (iPSC) harboring a *BMPR2* mutation, differentiated into monocytes (iMono) showed increased adhesion and transendothelial migration, independent of the mutation status of iEC. These findings highlight the potential of emerging therapies aimed at correcting abnormalities in both circulating monocytes and the vasculature.

## Methods

### PAH patient and control samples

All human samples used were coded and all patients and controls signed an informed consent under protocols approved by the Institutional Review Board on Human Subjects in Medical Research at Stanford University. We included PAH patients diagnosed with idiopathic PAH (IPAH) between the ages of 23 and 80 and healthy controls between the ages of 27 and 60. Whole blood from PAH patients was obtained from the Stanford Pulmonary Arterial Hypertension biobank (Stanford IRB #14083), and blood from healthy volunteers was obtained from the Stanford Biobank under the University sponsored precision health initiative (Stanford IRB #40869). Some experiments were conducted using de-identified blood from healthy individuals, obtained from the Stanford Blood Center (listed in Table E2 in the Online Data Supplement). iPSC used in the iMono and iEC experiments were derived by the Stanford CVI Biobank from de-identified skin fibroblasts provided by Dr. Eric Austin from Vanderbilt University, and by the PHBI Network investigators at Baylor College of Medicine, Vanderbilt University, and Allegheny hospital (Pittsburgh, PA). Control fibroblasts were donated and hereditary (H) PAH patient fibroblasts with a *BMPR2* mutation were collected with informed consent under an IRB approved at the procuring center. Patient’s characteristics, demographics, and hemodynamics and a list of the medications for each patient are shown in Table E1 in the Online Data Supplement. In Table E2 Stanford biobank control samples used for the scRNAseq had echocardiographic studies performed to exclude cardiovascular disease.

### Cell isolation and culture

#### Primary monocytes

PBMC were isolated by Ficoll-Paque (Histopaque™, Sigma Aldrich, St Louis, MO, Cat# 10771) from 15 mL of whole blood. Enriched monocytes were isolated from peripheral blood mononuclear cells (PBMC) via two methods: Magnetic bead isolation and FACS sorting.

#### THP1 cells

THP1 cells were obtained through ATCC (Cat#: TIB-202) and cultured according to ATCC standard protocol (ATCC website, protocol TIB-202 Product Sheet) in RPMI 1640 basal medium (Gibco, Cat#: 11875093) with 10% FBS, Penicillin-Streptomycin (10U/mL) (Gibco, Cat#: 15070063) and 2- mercaptoethanol (0.05mM) (Sigma, Cat#: M6250).

#### HEK293T cells

HEK293T cells were cultured in DMEM supplemented with 10% FBS and 1% penicillin-streptomycin.

#### PBMC FACS sorting and analysis

PBMC were resuspended in RPMI-1640 (Gibco, Dublin, Ireland) with 25mM HEPES, 2mM L-glutamine, and 100 I.U/mL penicillin and streptomycin (Gibco, ) and centrifuged at 400 g for 10 min. The pellet was resuspended in cold stain buffer (PBS (Invitrogen, Cat#: 10010023), 2 mM EDTA (EMD Millipore, Cat#: 15575020) and 5% BSA (Sigma, Cat#: A3059) and washed twice at 300 g at 4ºC. Cells were resuspended in cold stain buffer at 2 × 10^7^ cells/mL and aliquoted to the desired number of staining tubes. All cells were blocked with non-specific Fc-mediated (human IgG) interaction at 2.5 µg per 10^6^ cells for 10 min. Conjugated antibodies FITC-conjugated CD14 Cat#: 347493), PE-conjugated CD16 (Cat#: 347617), APC-conjugated CD19 (Cat#: 555415) (BD Biosciences, San Jose, California, USA), APC-conjugated CD56 (Cat#: 318310), APC-conjugated CD3 (Cat#: 300312), and Alexa-Flour 647-conjugated CD11c (Cat#: 301620) (BioLegend, San Diego, CA, USA), and ZombieNIR™ (BioLegend, Cat#: 423105) as a live/dead stain were added to appropriate tubes at manufacturers specified concentrations for 20 min at 4ºC, away from light. Monoclonal antibodies for surface antigen staining are listed in Table E3 in the Online Data Supplement. Following antibody incubation, cells were washed twice in cold stain buffer at 300 g for 2 min at 4ºC. Pellets were resuspended in a stain buffer, placed on ice, and immediately sorted on either FACS Aria Fusion, FACS Aria II or Influx (Beckman Coulter, Brea, CA, USA). Classical monocytes were sorted based on positive CD14 only; intermediate monocytes on CD14 and CD16 double positive; and non-classical monocytes on positive CD16. APC conjugated antibodies were used as a dump channel to eliminate other immune cells. For gating strategies, see Fig. E1 in the Online Data Supplement. FACS data were analyzed with FlowJo X 10.0.7r2 software (Tristar). Compensation was calculated using single-stained antibody-capture beads (CompBeads, BD Biosciences, Cat#: 552844). Fluorescence Minus One (FMO) control for each of these molecules were performed.

### Single cell RNA sequencing (scRNAseq)

PBMC were isolated as outlined above. Cells were diluted to 10^7^ cells/mL for optimal viability and taken to the Stanford Genomic Core for scRNAseq processing as per the 10X Genomics specified protocol, briefly outlined below.

#### Cell counting, GEM generation and barcoding

An aliquot of each cell suspension was stained with trypan blue. Concentration and viability were determined using the BioRad TC20 automated cell counter. Cells were counted for correct suspension volume for 5,000 cells total. Following addition of the 10X Master Mix™ (real time reagent mix, real time primer, Additive A, and real time enzyme mix) to the Single Cell A Chip, cells were loaded into the same well. Single Cell Gel Bead suspension was added in a separate well on the chip, as well as the partitioning oil in a third separate well. The chip is covered with a gasket and loaded into the 10X Genomics Chromium™ Controller. Following the completion of the run, the gel beads in emulsion (GEMs) are transferred into a PCR tube for amplification.

#### Library preparation, and sequencing

Single-cell libraries were prepared using the 10X Genomics Chromium 3’ Gene Expression Solution. The average fragment size of each completed library was determined using the Fragment Analyzer (Agilent). Final library concentration was determined using the Qubit High Sensitivity double stranded (ds)DNA Assay (ThermoFisher, Cat# Q32850). Libraries were normalized to a common concentration prior to being pooled and sequenced on the Illumina HiSeq-4000.

#### Single cell transcriptome sequence pre-processing

Sequencing reads were demultiplexed and mapped to the Homo sapiens genome assembly GRCh38 using software Cell Ranger V3.0 (10X Genomics). Mapped reads were aggregated into an expression matrix by Cell Ranger V3.0 (10X Genomics). This matrix was loaded to R and a Seurat object was created using package “Seurat” V4.0.0. 40,029 cells were obtained for quality control. Doublets were removed by removing cells whose nFeature < 0.22*nCount– 170. For samples using chemistry V2, cells with less than 13% mitochondrial genes and 2,100 unique genes with more than 1,100 reads were kept for downstream analysis. For samples using chemistry V3, cells with less than 20% of mitochondrial genes and 3,500 unique genes, with more than 2,100 reads were kept for downstream analysis. Cells expressing less than 50 housekeeping genes according to a relevant search [16] were removed. 35,557 samples carrying 20,746 unique features were preserved for downstream analysis.

#### ScRNAseq statistical analysis

Clean data were transformed and aggregated using sct transform following standard protocol [17]. Briefly, the two batches (V2 chemistry and V3 chemistry) were integrated based on 3,000 selected features across all samples. Principal Components Analysis (PCA) was performed based on 2,000 most variable genes within the integrated dataset. Following examination of variability within the dataset, uniform manifold approximation and projection (UMAP) based on 30 principal components was applied for visualizing cells in two dimensions. We implemented the Seurat package to identify raw cell clusters, calling 28 clusters at a resolution of 1.3 through the shared nearest neighbor (SNN) modularity optimization-based clustering algorithm. Representative gene markers of each cluster were calculated by building a logistic regression model for each unique gene in one cluster and then comparing this with the null model with a likelihood ratio test. For downstream analyses, two clusters were excluded as they did not represent peripheral blood mononuclear cells (PBMC). Additionally, four clusters were specifically omitted from Figures E1B-C in the Online Data Supplement, as they lacked distinct gene markers discernible at the chosen resolution after marker calculation.

Monocytes/Conventional Dendritic Cell clusters were identified as described in Results section. Cluster specific differential abundant genes between controls and PAH patients was performed based on modeling each gene by negative binomial distribution and comparing the distribution between cohorts using DESeq2 [18]. Gene Ontology analysis was performed using R package “clusterProfiler” [19]. Briefly, genes from DEG analysis are selected at absolute log_2_ fold change > 0.3 and adjusted *P* value < 0.05. This list of genes was mapped with gene sets from R package “org.Hs.eg.db.” Single cell trajectories were calculated by R package “Monocle 3” [20]. Briefly, samples from control and PAH patients are normalized to 2,104 cells. Projections of cell trajectories was performed on the UMAP result. The start point of the trajectory was set on the area of CD14 farthest to the CD16 positive monocytes cluster based on its previously reported natural development progress [21]. If not specifically mentioned, the Figure was then produced by R package ggplot2. Detailed package version and R environment information can be found at https://github.com/xzhou7/scRNA_PBMC/blob/main/R.Session.Info.

### Primary monocyte culture for cell death assay

Following isolation by FACS sorting, classical monocytes were cultured overnight in RPM1-1640 with 10% fetal bovine serum (FBS) (Gibco) in 24-well plates (Corning, Corning, NY, USA) at 1 × 10^5^ cells/well. These classical monocytes were stimulated with GM-CSF (10ng/mL; Prepotech, Rocky Hill, NJ, USA, Cat#300-03), m-CSF (10 ng/mL; Prepotech, Cat#300−25) and low-dose Lipopolysaccharides from Escherichia coli O111:B4 (LPS) (5 pg/mL; Company, Cat#L4391) for 24 h. All cells were collected and centrifuged at 400 x g and processed for FACS analysis as outlined in the “PBMC FACS Sorting and Analysis” methods section, including a live/dead stain using ZombieNIR™ (Biolegend).

### Real time PCR (RT qPCR) of primary monocytes and THP1 cells

Total RNA was isolated using Trizol TRI-Reagent (Sigma, Cat# T9424). The quantity and quality of RNA was determined by using a spectrophotometer. RNA was reverse transcribed using the High-Capacity RNA to cDNA Kit (Applied Biosystems, Foster City, CA, Cat# 4387406). Total RNA (2 µg) from each sample was assayed in triplicate for each reverse transcription (20 µL volume). RT-qPCR was performed using 2µL cDNA per sample, 1 µL mixed primers (5 µM), 5 µL Power SYBR green PCR Master Mix (Applied Biosystems, Cat# 4368577), 2 µL ddH_2_O. Each measurement was conducted in triplicate using a CFX384 Real Time System (BioRad) in a 10 µL reaction. The PCR conditions were pre-denaturation at 95℃ for 3 min, followed by 40 cycles of 95℃ for 15 s, annealing and extension at 56℃ for 60 s, or depending on the primers to adjust annealing temperature. The mRNA relative transcriptional level was calculated by the relative quantitative method 2 − ΔΔCt with the formula: ΔΔCt = ΔCt (experimental group) −ΔCt (normal group), ΔCt = Ct (target gene) − Ct (internal reference), and 2 − ΔΔCt represented the mRNA relative transcriptional level. Expression levels of selected genes were normalized to beta 2 microglobulin (B2M) mRNA. Primers sequences were designed using PrimerBank (http://pga.mgh.harvard.edu) and are listed in Table E5 in the Online Data Supplement.

### SiRNA BMPR2 knockdown in THP1 cell line

THP1 cells at a density of 5 × 10^5^ cells/mL were cultured overnight in a 6-well plate, followed by siRNA transfection with siRNA *BMPR2* (SMARTpool ON-TARGETplus, Origene, Rockville, MD, Cat#: L-001230-00-005) or siRNA Control (ON-TARGETplus non-targeting Control siRNA, Origene, Cat#: D-001230-01) using the TransIT-TKO reagent (Mirus, Madison, WI Cat# MIR2150) according to the manufacturer’s recommendations. BMPR2 protein reduction was confirmed in membrane fractions as part of a separate study [data not shown].”

### Lentiviral transfection of THP-1 cell line

HEK293T cells were sourced from ATCC (Cat#: CRL-3216) and maintained in Dulbecco Modified Eagle Medium (DMEM) High Glucose (Gibco, Cat#: 11965092) supplemented with 10% FBS and 1% penicillin-streptomycin, following ATCC standard protocol. The cells were seeded at a density of 5 × 10^5^ cells/mL in a T75 flask and incubated overnight. The next day, the old media was removed and replaced with 8 mL of fresh medium. This was subsequently treated with 2 mL of reduced serum medium (Opti-MEM, Gibco, Cat#: 31985070) pre-incubated for 15 min with 7 µg of either scramble shRNA (Cat#: VB010000-0009mxc) or BMPR2 shRNA plasmids (Cat#: VB900034-4780ezd) (Vector Builder Chicago, IL), 7 µg of lentiviral packaging plasmid mix (Celecta, Mountain View, CA, Cat#: CPCP-K2A), 30 µL Lipofectamine 3000, and 14 µL P3000 reagent (Invitrogen, Cat# L3000001). After a 24-hour incubation, green fluorescence was observed, and the media were collected. The collected media were then filtered through a 0.45 μm filter to remove cellular debris.

THP1 cells (Cat#: TIB-202) were cultured according to ATCC standard protocol in RPMI 1640 basal medium (Gibco, Grand Island, NY, Cat#: 11875093) with 10% FBS, Penicillin-Streptomycin (10 U/mL) and 2-mercaptoethanol (0.05 mM) (Sigma, Cat#: M6250). Before transduction, THP-1 cells were plated in 6-well plates and treated with monocyte attachment medium (PromoCell, Heidelberg, Germany, Cat#: C28051) for one hour to enhance attachment and transfection efficiency. The filtered lentiviral media from HEK293T cells was added to the THP-1 cells, and polybrene (Santa Cruz Biotechnology, Cat# CAS 28728-55-4) at a concentration of 10 µg/mL was included to improve transduction efficiency. The cells were incubated with the viral media for 72 h, followed by puromycin selection at 1 µg/mL in fresh media for 5–7 days until non-transduced cells were eliminated. Transduction efficiency was initially assessed by observing GFP expression under a fluorescence microscope. To confirm *BMPR2* knockdown, RNA was extracted, and qRT-PCR was performed to measure *BMPR2* mRNA levels, normalized to *B2M*.

### Generation of iMonocytes

#### iMono differentiation studies

The subject-specific induced pluripotent stem cells (iPSC) were generated from peripheral blood cells of patients and from healthy volunteers (control) who did not have the *BMPR2* mutation as previously described [22]. All iPSC lines assessed had the potential of unlimited self-renewal and the ability of three germ layers differentiation. This was tested by performing a monolayer differentiation assay which spontaneously drives the cells towards the three germ layers in vitro. We determined the marker gene expression for the mesoderm (*HAND1*), endoderm (*SOX17*), and ectoderm (*PAX6*) by RT-qPCR as per our previous publications [23]. A stepwise protocol was developed to drive iPSC differentiation into mesoderm precursors under feeder-free and chemical defined conditions, followed by their specific lineage commitment and maturation [24]. Briefly, iPSCs were seeded onto Matrigel-coated plates at ~ 8 mg/mL (BD Biosciences, Cat# 354230) at approximately 10 ~ 30 cells per colony, with Rock inhibitor Y-27,623 (10 nM, Tocris Bioscience, Bristol, UK, Cat# 1254). Step 1: After 24 h of recovery in Essential 8™ Medium (Gibco, Cat# A1517001), cells were differentiated into mesoderm progenitors in differentiation medium STEMdiff™ Apel™2 media (Stemcell Technologies, Vancouver, BC, Canada, Cat# 05275) with Penicillin/streptomycin (50 U Penicillin G/ 50 mg streptomycin sulfate; Invitrogen). During the first two days of culture, Apel™2 was supplemented with recombinant human (rh) vascular endothelial growth factor (rhVEGF, 10 ng/mL; Gibco, Cat# PHC9394), rh Bone morphogenetic protein 4 (rhBMP4, 10 ng/mL; R&D systems, Cat# 314-BP-050), and rh Basic fibroblast growth factor (rhbFGF, 10 ng/mL; PeproTech, Cranbury, UK, Cat# 100-18B). From day 3 to day 7 (Step 2), additional supplement of hematopoietic stem/progenitor cells (HSPC) growth factors and cytokines: rh stem cell factor (rhSCF, 50 ng/ml; PeproTech, Cat# 2830), rh Flt-3 Ligand (rhFlt-3 L, 50 ng/mL; PeproTech, Cat# 2941), and rh Thrombopoietin (rhTPO, 50ng/mL; PeproTech, Cat# 02720). Step 3 (day 7–10), rh Granulocyte-Macrophage Colony-Stimulating Factor (rhGM-CSF, 100 ng/mL; PeproTech, Cat# 300-03) was added to the culture medium. During step 4 (from day 10–14), the culture medium was changed to RPMI 1640 with 10% of FBS (R&D Systems, Cat# S11150H) plus rhFlt-3 ligand, rhTPO, rhSCF, and rhGM-CSF to further promote differentiation into myeloid lineage cells. After 14 days of induction (Step 5), in order to enrich monocyte generation from myeloid lineage cells, the cells in suspension were collected, seeded into regular cell culture dishes and cultured for an 5 additional days in RPMI with 10% of FBS, supplemented with rhGM-CSF and rh Macrophage Colony-Stimulating Factor (rhM-CSF, 100 ng/mL; PeproTech, Cat# 300 − 25), the key regulators of monocyte proliferation and differentiation [25].

#### iMono validation

For the flow cytometric immunophenotyping of iPSCs and monocytes, the suspension cells were harvested at designed time points. Following washing with PBS, cells were stained for 30 min in the dark at 4 °C with the following anti-human monoclonal antibodies: PE-conjugated CD34 (BioLegend, Cat#: 343606), APC-conjugated CD45 (BioLegend, Cat#: 304012), FITC-conjugated CD14 (BioLegend, Cat#: 325604), APC-conjugated CD16 (BioLegend, Cat#: 302012), and PE/Cy5-conjugated CD11b (BioLegend, Cat#: 301308). After washing with PBS, stained cells were resuspended and analyzed by fluorescence-activated cell sorting (FACS) (MACS Quant, Miltenyi Biotec). (Gating strategy is shown in Online Data Supplement Figure E2B). Monoclonal antibodies for surface antigen staining are listed in Table E4 in the Online Data Supplement. This protocol yields surface expression similar to plasma monocytes [23].

### Generation of iEC for iMono functional studies

#### Induced endothelial cell (iEC) differentiation studies

The iPSC lines used for iMono differentiation were also differentiated into endothelial cells (iEC) following our previously published protocol [22]. iPSCs were seeded onto Matrigel-coated plates at ~ 8 mg/mL (BD Biosciences) at approximately 10 ~ 30 cells per colony, with Rock inhibitor Y-27,623 (10 nM, Tocris Bioscience, Cat# 1254). After 24 h of recovery in Essential 8™ Medium (Gibco, Cat# A1517001), cells were differentiated into mesoderm progenitors in differentiation medium STEMdiff™ Apel™2 media (Stemcell Technologies, Vancouver, BC, Canada, Cat# 05275) with Penicillin/streptomycin (50U Penicillin G / 50 mg streptomycin sulfate; Invitrogen, Cat# 15140122) and supplemented with rhVEGF, (10 ng/mL; Gibco, Cat#: PHC9394), rhBMP4 (10 ng/mL; R&D systems, Cat#: 314-BP-050) and rhbFGF (10 ng/mL; PeproTech, Cat#: 100-18B) for 7 days. Cells were lifted using TrypLE TM Express Enzyme (Invitrogen, Cat# 12563), and assessed for CD31 and CD34 surface antigen expression via FACS. Subsequently, CD31 positive cells from the induction culture were enriched using CD31 human Magnetic MircoBead Kit (Miltenyi, Cat# 130-091-935), and to promote further expansion [26], iECs were further cultured on Collagen I coated plates (Corning BioCoat, Cat# 354450) with endothelial culture medium, 1:1 EGM™2 supplemented with 5% FBS, hydrocortisone, hFGF-B, VEGF, R3-IGF-1, Ascorbic Acid, hEGF, GA-1000, Heparin- as per manufacturer’s instructions (Lonza, Basel, Switzerland, Cat# CC-3162) and StemPro™-34 SFM (Gibco, Cat# 10639011).

***Acetylated low density lipoprotein (LDL) uptake iEC validation assay*** was performed by incubating the iEC with 10µg/mL acetylated LDL (Ac-LDL) labeled with 1,1’-dioctadecyl-3,3,3’,3’-tetramethylindo-carbocyanine perchlorate (DiI-Ac-LDL) (Invitrogen, Cat# L3484) for 4 h at 37 °C. The DiI-Ac-LDL uptake was assessed and quantified by fluorescent microscopy. Subsequently, cells were dissociated into single cells with TrypLE and quantified via FACS. Data acquisition was performed on a MACSQuant Flow Cytometer (Miltenyi Biotec, Bergisch Gladbach, Germany) and the results were analyzed with FlowJo software (FlowJo, LLC). iEC in passage 3 were used for all subsequent experiments.

### In vitro co-culture

Experiments were performed following our previously published protocols [27].

#### Transmigration assay

Transwell inserts with transparent polyester (PET) membrane (3.0 μm pore size) (Corning, Cat# 353096) of a 24-well plate were coated with human fibronectin (31.25 µg/mL) (Corning, #54008), to minimize cytokine production and ensure greater adherence, for 30 min. iEC were then seeded at a density of 60,000 cells/well in the upper insert. After iEC formed a confluent monolayer (24 h), control or HPAH iMono at a 10:1 iMono/iEC ratio were added to the top insert with 1:1 ratio mixed medium (iEC media/RPMI with 10% FBS). The same medium was only added to the bottom chamber. The iMono that transmigrated to the bottom chamber were collected after 24 h co-culture. Cells were counted with BioRad TC20 automated cell counter and expressed as cells / mL.

#### Adhesion assay

iEC were seeded in a 24-well plate coated with fibronectin (31.25 µg/mL) at 1 × 10^5^ cells/well. After iEC formed a confluent monolayer (24 h), control or HPAH iMono were added at a 10:1 iMono/iEC ratio with 1:1 ratio mixed medium (iEC media/RPMI with 10% FBS). Following 24 h in co-culture, cells were washed with PBS and adhered iMono were counted via DAPI staining using their distinct smaller nuclei on the endothelial monolayer. Cells were imaged using a Zeiss inverted fluorescence microscope using Zen 3.3 imaging software (Zeiss, Oberkochen, Baden-Württemberg, Germany). Quantification was conducted on 10 fields of view on 3 technical replicates for 3 biological replicates.

#### iEC immunofluorescence staining

Cells were co-cultured with control or HPAH iMono at 10:1 iMono/iEC ratio for 24 h then fixed with 4% paraformaldehyde for 10 min at room temperature. The primary antibody mouse anti-VE-cadherin (CDH5) (1:100 Santa Cruz, CA, Cat# sc-9989) and rabbit anti-intercellular adhesion molecule (ICAM1) (1:100, Abcam, Cat# ab109361) were applied overnight at 4^o^C, followed by secondary antibody goat anti-rabbit-Alexa Flour 594 and donkey anti-mouse-Alexa Flour 488 (1:1000, Invitrogen, Cat# a11012 & a21202 respectively) for one hour at room temperature. DAPI (1:10,000, Thermo, Cat# D1306) was used for cell nuclei detection. Cells were imaged using a Zeiss inverted fluorescence microscope using Zen 3.3 imaging software (Zeiss).

### Animal studies

The Animal Care Committee at Stanford University approved all the experimental protocols used in this study in accordance with the guidelines of the American Physiological Society.

#### *Cx3cr1Cre/Td/Bmpr2*^*−/−*^*Mice*

*Bmpr2*^flox/flox^ mice were generated in our lab and were crossed with *Cx3cr1-Cre* and Td tomato mice (Stock No: 020940 and 007914, The Jackson Laboratory, Bar Harbor, MA). Further breeding of the offspring generated *Cx3cr1Cre/Td/Bmpr2*^*−/−*^ (KO) progeny. The mice were initially of a mixed C57 BL/6J, SV129 and FVB background but were backcrossed for more than six generations onto a C57BL/6J background.

The inducible KO and control mice at age of 6–8 weeks were fed a tamoxifen containing diet (ENVIGO, product code: TD.130859, 400 mg/Kg food) for four weeks to induce deletion of target genes in monocytes before exposure to hypoxia only as described below. The four-week tamoxifen diet was well tolerated with no toxicity. Isolated peripheral blood monocytes and freshly isolated lungs were checked for Td tomato expression with co-expression of LY6C and CD68, respectively. Each experiment included 5 mice per sex per group. The results show 3–5 mice per sex, per group.

#### Genotyping by polymerase chain reaction (PCR)

We followed the protocol provided by The Jackson Laboratory (https://www.jax.org/Protocol) for detection of transgene, *Cx3cr1-Cre* (Jax website, stock No. 020940) and Td tomato (Jax website, stock No. 007914). For *Bmpr2* transgene expression, tails were collected and processed with Mouse Tail DNA Extraction kit (101 Bio, Mountain View, CA, Cat# T605). Five µL of tissue lysate containing 50–100 ng of genomic DNA were used for each PCR reaction. 1.5-2% agarose gel electrophoresis was performed, and gels were analyzed by Image Lab (BioRad, Hercules, California). PCR Primers used for genotyping are listed in Table E5 in the Online Data Supplement.

#### Hypoxia exposure

The inducible KO mice after the tamoxifen diet, or *Cx3cr1Cre/Td* as controls were placed in a hypoxia chamber (BioSpherix, Lacna, NY, Cat#. A-30274-P) and exposed to 10% inspired O_2_ with access to food and water for three weeks. Mice and O_2_ status were monitored daily.

#### Two-dimensional echocardiographic assessments

Prior to the hemodynamic assessment, two dimensional echocardiographic measurements of cardiac function were carried out with the GE Vivid7 and GE Vivid95 ultrasound machines in unventilated mice under isoflurane anesthesia (1.5 − 2.5% in 2 L O_2_/min). Mice chests were shaven, and echocardiographic images were used to determine the heart rate (HR) and the pulmonary arterial acceleration time (PAAT). Mice were monitored during the echocardiographic assessment.

#### Hemodynamic assessments

Cardiac catheter measurements of the right ventricular systolic pressure (RVSP) were carried out in unventilated mice under isoflurane anesthesia (1.5 − 2.5% in 2 L O_2_/min) with closed chests using a 1.4 Fr Millar catheter (Millar Instrument, Houston TX, Model SPR-671), inserted through the jugular vein, and recorded with the PowerLab 7 system (AD Instruments, Model PowerLab 7, Colorado Springs CO) as previously described [28].

#### Tissue processing

Mice were sacrificed by cervical dislocation under anesthesia, and the heart and lungs were harvested after flushing the pulmonary circulation with cold PBS. The lungs were fixed with 10% v/v neutral buffered formalin (NBF), embedded in paraffin for sectioning, and routine histology and morphometric analyses conducted as previously described [28]. Right ventricular hypertrophy (RVH) was assessed as the dry weight of the right ventricle (RV) relative to the weight of the left ventricle plus septum (LV + S).

#### Blood collection and monocyte isolation

Mice were humanely sacrificed and immediately opened in the peroneal space to withdraw blood from the superior vena cava. Mouse blood was pooled from 3 mice for subsequent PBMC isolation using Ficoll-Paque as outlined above. Monocytes were isolated using the Monocyte Isolation Kit (BM) for Mice (Miltenyi, Cat#. 130-100-629) and processed for RT-qPCR as outlined above.

#### Mouse bone marrow isolation and bone marrow derived macrophage (mBMDM) differentiation

Mice were humanely sacrificed by cervical dislocation, and both the tibia and femur of both legs were surgically removed and flushed with Eagle’s Minimum Essential Medium (EMEM, Sigma Aldrich, Cat#: M4655) + 10% FBS with Penicillin-Streptomycin (10 U/mL) (Gibco) medium to collect all bone marrow. This was filtered and strained to achieve a single cell suspension and plated in L929 conditioned media for 2 days. mBMDM culture was washed with PBS on day 2 to eliminate bone marrow debris and dead cells and cultured for a further 5 days in EMEM + 10% FBS with Penicillin-Streptomycin (10 U/mL) (Gibco).

#### Identification and quantification of Td + Cells

Paraffin-embedded mouse lung tissue sections were deparaffinized and rehydrated following a standard protocol. Sections were permeabilized (0.2% Triton X-100 in 1X TBS) for one hour at room temperature, followed by 3 × 5 min wash in in ddH_2_0 and mounted with ProLong™ Gold Antifade Mountant with DAPI (Invitrogen, Cat#: P36931). Images were acquired using a SPOT imaging system (SPOT IMAGING, Sterling Heights Headquarters, MI) connected to a Leica microscope (JH Technologies, Leica DMLB model, Fremont, CA) with 10X and 20X objectives. Quantification was performed on 5 fields of view (FOV) of the distal pulmonary arteries per mouse/ group, where only perivascular macrophages were counted using Fiji ImageJ2, Version 2.9.0/1.53t.

### Statistical analysis

Monocytes/Conventional Dendritic Cell clusters were identified as described in Results section. Individual data points are shown, with bars representing mean ± standard error of the mean (SEM). We were limited by the number of experiments that could be done using freshly isolated monocytes, so in general the n in these studies is lower than in those where we could use frozen PBMC to isolate the monocytes. Data with a sample size *n* < 8 was analyzed using the Mann-Whitney non-parametric test. Samples with n of 3 are confirmatory of other assays or represent technical replicates from cell lines, pooled biological samples where specified (mouse studies), or biological replicates with 3 technical replicates for each biological replicate (iMono studies). In some of the figures, the average of the control values is set as one and the fold change of each individual control value is shown to reflect variance, and for the test samples, the fold change is shown for each determination, relative to the average of the control cohort. Statistical analysis was performed using GraphPad Prism (v8.4.1).

## Results

### IPAH and control IPAH monocyte clusters overlap

**Fig. 1:**
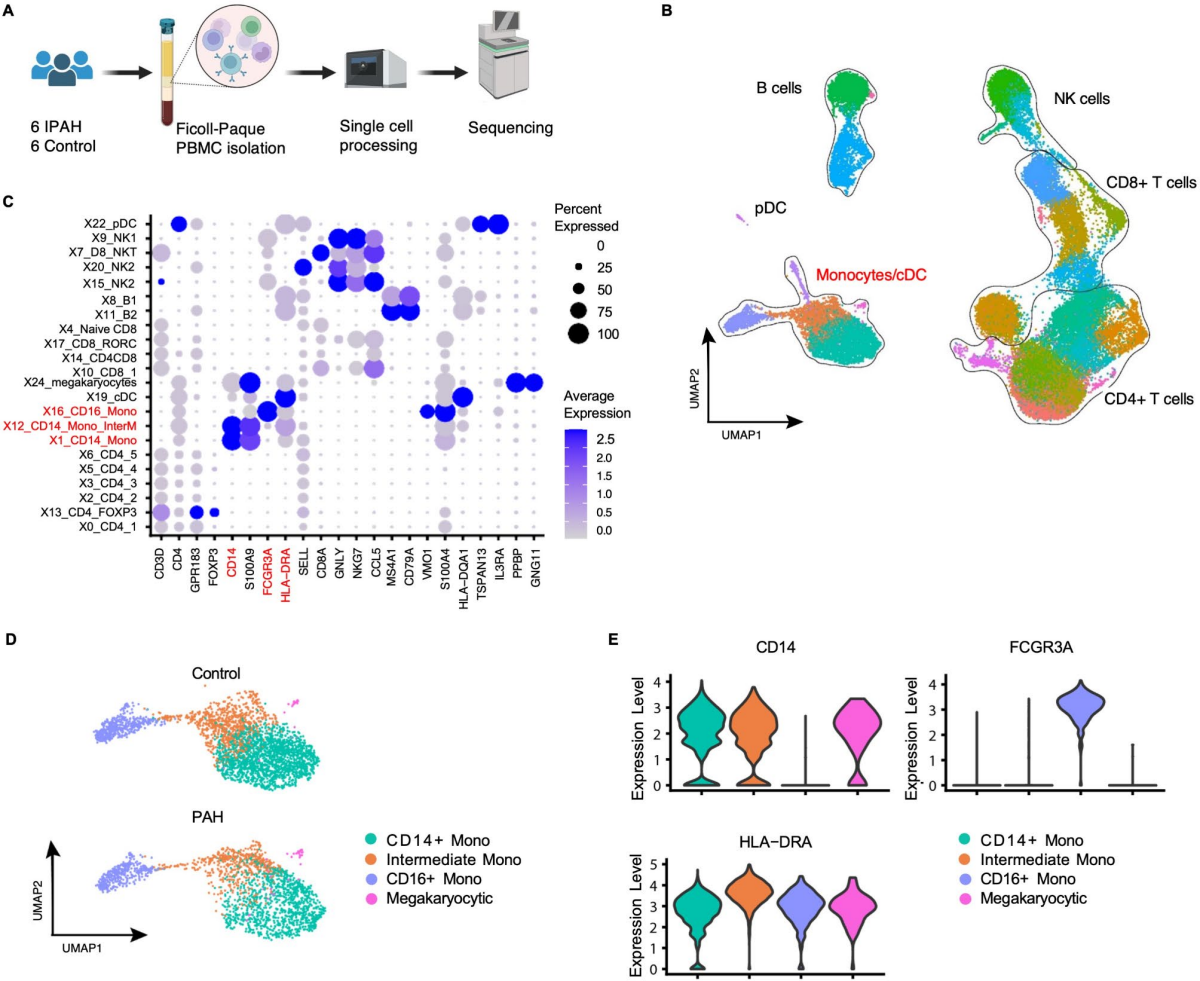
IPAH and Control monocyte clusters overlap. Peripheral blood mononuclear cells (PBMC) were isolated from peripheral blood of healthy donors (Controls) and idiopathic (I) PAH patients as described in the Methods. **(A)** Schematic outline of the study design. PBMC were isolated from whole blood of 6 IPAH patients and 6 Controls and processed for single cell sequencing using the 10X Genomics pipeline. Schematic constructed using Biorender.com. **(B)** UMAP of Control and IPAH PBMC. Major cell types - natural killer (NK) cells, CD4^+^ and CD8^+^ T cells, monocytes/ conventional dendritic cells (cDC) and B cells– are grouped with a black outline. **(C)** Dot plot of gene markers for PBMC cell populations **(D)** Monocyte subpopulation identification using CD14, CD16 (FCGR3A) and HLA-DRA gene expression. **(E)** Violin plot CD14, FCGR3A (CD16) and HLA-DRA expression in monocyte clusters

scRNAseq was applied to unstimulated PBMC of 6 IPAH and 6 control subjects (Fig. 1A). Demographic and phenotypic data of samples related to this, and subsequent experiments are shown in Tables E1 and E2 in the Online Data Supplement. PBMC were processed using a combination of 10X Genomics v2 or v3 chemical reagents (Fig. 1A). Following sequencing, data handling, and filtering out low quality cells, we obtained the transcriptome from 35,521 PBMC (IPAH = 14,273 cells, and control = 21,248 cells). Unsupervised clustering identified 26 immune cell clusters (Fig. 1B) and IPAH and control samples are overlapping (Figure E1A-B in the Online Data Supplement). Monocyte/dendritic cells (cDC), CD8^+^ T cells, CD4^+^ T cells, B cells, plasmacytoid dendritic cells (pDC) and natural killer (NK) cells were further classified using established markers for each cell type (Figure E1C). Monocytes identified using *CD14*, *FCGR3A* (CD16) and *HLA-DRA*, were classified into classical (CD14^+^/CD16^−^), intermediate (CD14^+^/CD16^+^), and non-classical (CD14^−^/CD16^+^) (Fig. 1D, E) and are the focus of this manuscript.

### IPAH monocyte differentially expressed genes (DEG) reveal a CD14^low^/STAT1^high^ signature

**Fig. 2:**
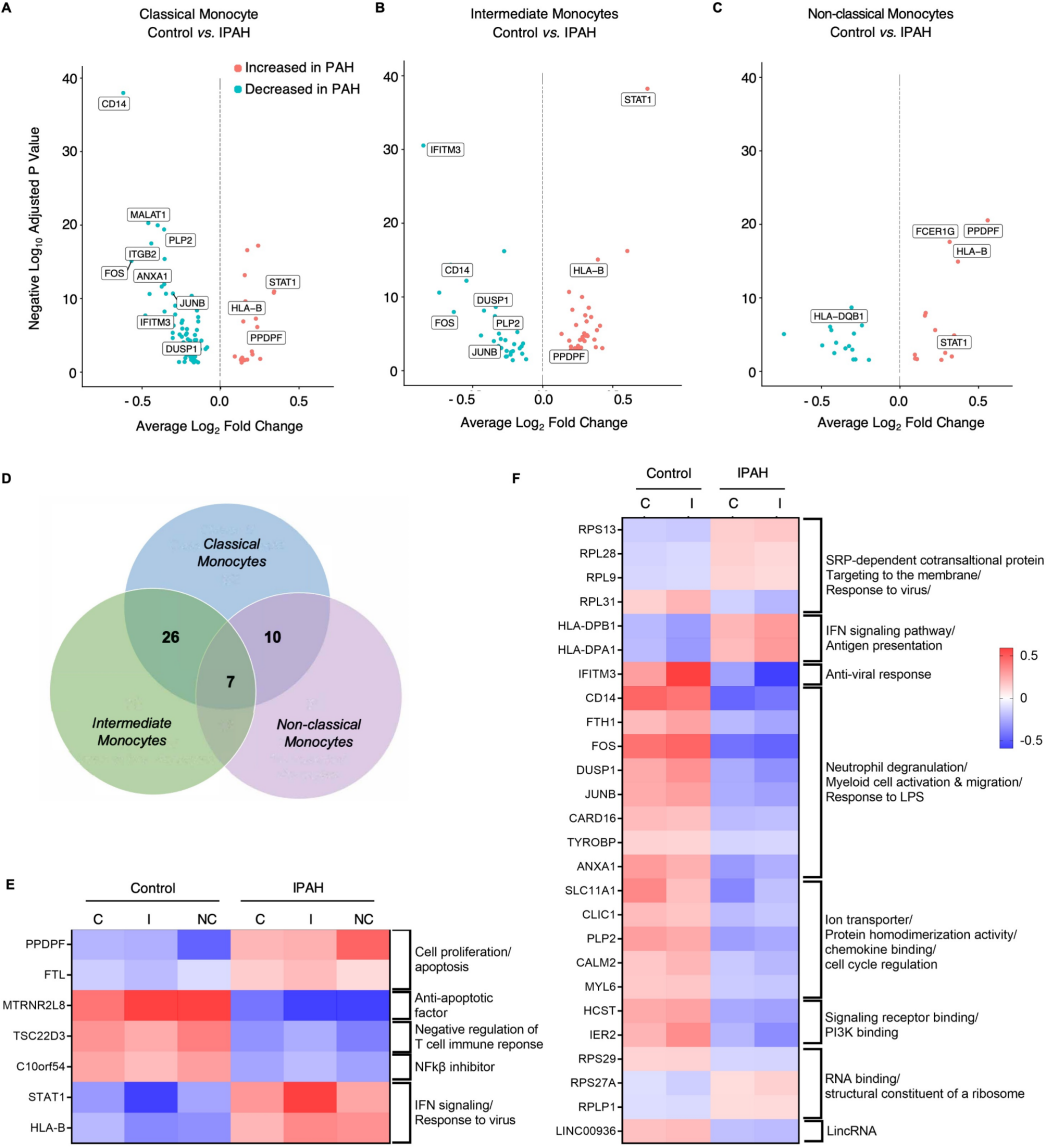
IPAH monocyte differentially expressed genes (DEG) reveal a CD14^low^/STAT1^high^ signature. Differential expression (DE) analysis was performed on Control vs. IPAH classical, intermediate, and non-classical. **(A-C)** Volcano plots of DE genes, comparing fold change (FC) in genes up and down regulated in Control vs. IPAH. Orange dots represent upregulated genes in IPAH monocytes (adjusted *p* < 0.01 and FC ≥ 2), blue dots represent downregulated genes in IPAH monocytes (*p* < 0.01 and FC ≤ 0.5). **(D)** Venn diagram showing number of DE genes in each monocyte subpopulation, identifying common DE genes for all three subpopulations in IPAH compared to Control. **(E)** Heat map of individual DE genes and molecular pathway each gene is implicated in, for the DE genes common to all three monocytes subpopulations. **(F)** Heat map of individual DE genes and molecular pathway each gene is implicated in, for the 26 DE genes common to the classical and intermediate monocytes subpopulations. *n* = 6 IPAH patients or Controls. In (**E** and **F**), C: classical, I, intermediate, and NC, non-classical monocytes

We performed a pairwise comparison of differentially expressed (DE) genes, identifying seven DE genes between IPAH and controls for each monocyte cluster (Fig. 2A-C). *HLA-B* and *STAT1* were upregulated across all IPAH monocyte populations, consistent with activation of the interferon (IFN) signaling pathway [29]. This aligns with our previous observation of elevated retroviral HERV-K elements in PAH monocytes that induce an IFN response related to double stranded DNA and RNA [29, 30]. Five additional genes were differentially regulated across all three monocyte subpopulations (Fig. 2D); pancreatic progenitor cell differentiation and proliferation factor (*PPDPF)*, ferritin light chain (*FTL)*, both associated with cell differentiation, were increased in IPAH vs. control monocytes. Conversely, genes that were downregulated included MT-RNR2 Like 8 *(MTRNR2L8)*, a non-coding RNA implicated as an anti-apoptotic factor [31], TSC22 domain family member 3 (*TSC22D3*), a gene suppressed by type 1 IFN [32], and *C10orf54*, a monocyte activation agonist [33] that suppresses T cell activation [34] (Fig. 2E).

The top 20 DE genes in both classical and intermediate monocytes revealed decreased inflammatory response genes in IPAH vs. control samples that included *CD14*,* FOS*, *JUNB*, dual specificity phosphatase 1 *(DUSP1)*, proteolipid protein 2 *(PLP2)*, and annexin A10 (*ANXA10*), as well as interferon induced transmembrane protein 3 (*IFITM3*) as an anti-viral response (Fig. 2A-B and F). Other top 20 DE genes identified play a role in activation, cell arrangement, cell adhesion and survival in monocytes (Fig. 2F) [35, 36]. These data suggest two distinct IPAH monocyte phenotypes: one characterized by CD14^low^ classical and intermediate monocytes, and the other marked by STAT1^high^ expression across all three monocyte populations.

### IPAH classical and intermediate monocyte subpopulations show a deviated differentiation trajectory

**Fig. 3:**
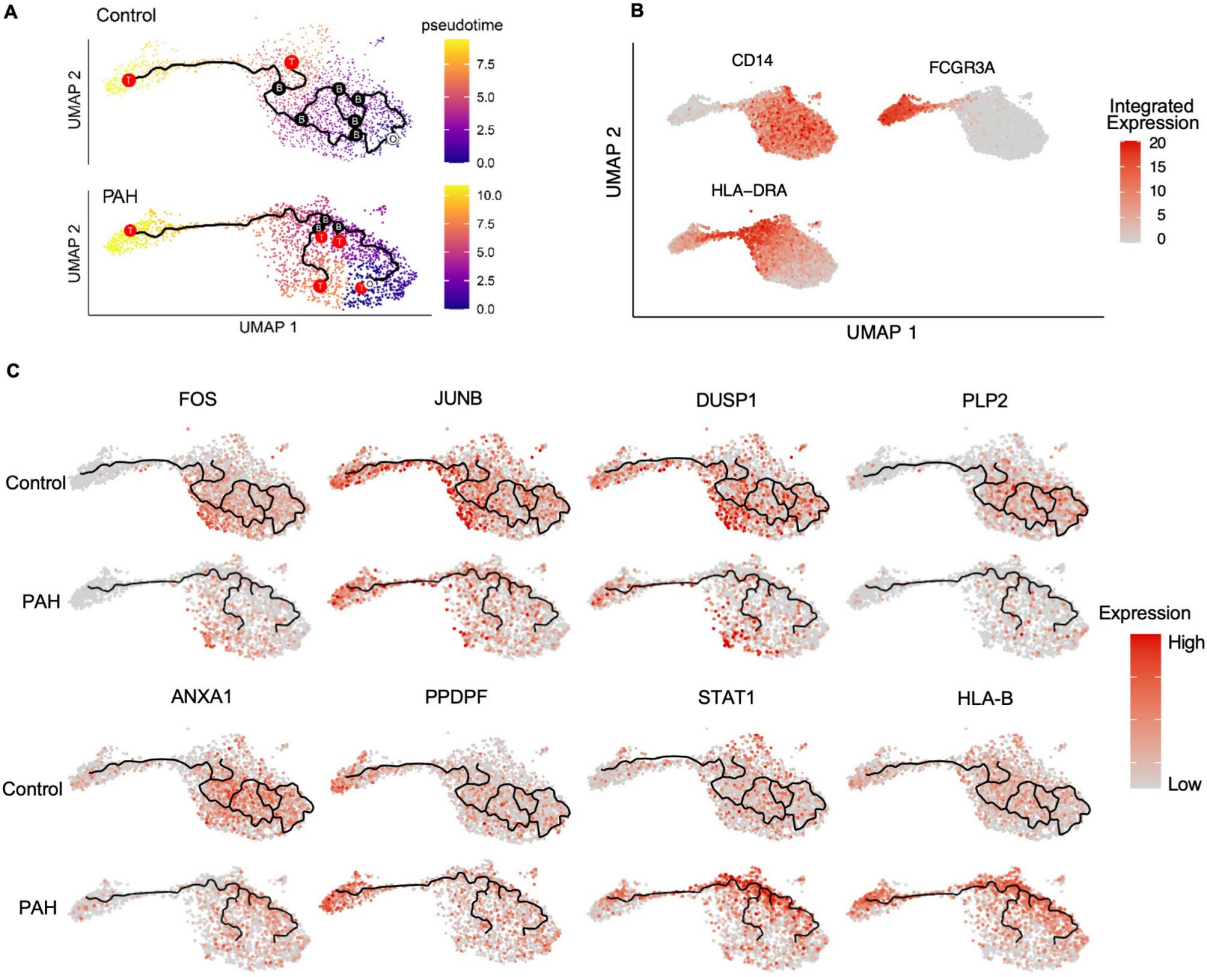
IPAH classical and intermediate monocyte subpopulations show a deviated trajectory. **(A)** Trajectory analysis (black line) for Control and IPAH monocytes. White circled ”O”” represents the starting pseudotime, red circled “T” represent terminal endpoints, and black circled “B” represent branching of trajectory. Pseudotime color spectrum shows purple as the starting time, progressing to yellow as the endpoint. **(B)** UMAP of monocyte clusters configured for trajectory analysis showing subpopulation characterization markers: Classical monocytes, high *CD14* expression; Intermediate monocytes, high *HLA-DRA* expression; non-classical monocytes, low *CD14* and high *FCGR3A* expression. **(C)** UMAP of gene expression profile for: *FOS*,* JUNB*,* DUSP1*,* PLP2*,* ANXA1*,* PPDPF*,* STAT1* and *HLA-B*. *n* = 6/ group

Next, to investigate the basis of these two IPAH monocyte phenotypes, we performed a pseudo-time trajectory analysis. Pseudotime color spectrum shows purple as the starting time, progressing to yellow as the endpoint (Fig. 3A). Monocyte sub-populations were identified by their transcriptome using CD14 (classical), HLA-DRA (intermediate) and non-classical (FCGR3A) markers (Fig. 3B). Control monocytes originated as *CD14*^hi^ classicals (white circle “O”, Fig. 3A), and progressed along multiple trajectories toward intermediate (*HLA-DR*) and non-classical (*CD16*) phenotypes, as determined by gene expression, with six branching nodes (black circle ”B”, Fig. 3A). Two terminal ends were observed (red circle “T”, Fig. 3A), one in the intermediate cluster and the other in the non-classical cluster (Fig. 3A). Additionally, the high expression of *FOS*,* JUNB*,* DUSP1*,* PLP2* and *ANXA10* in the control classical monocyte cluster (Fig. 3C) illustrates that these activation genes are important in maintaining classical monocyte survival and maturation, consistent with previous studies [12, 37].

In contrast, IPAH monocytes exhibit restricted trajectories with an increase in terminal ends (red “T”, Fig. 3A). Starting from the same point as the control group (white ”O”, Fig. 3A), IPAH cells terminally differentiate into non-classical monocytes (red “T”), immature intermediate monocytes (red “T”), or classical monocytes (red “T”) in the regions of low *FOS*,* JUNB*,* DUSP1*,* PLP2* and *ANXA10* (Fig. 3C). These findings are consistent with impaired survival in IPAH classical and intermediate monocytes with decreased CD14 expression and diminished immune activation.

Elevated *STAT1*, *PPDPF* and *HLA-B* expression were observed in intermediate and non-classical IPAH monocytes that mature and differentiate into non-classical monocytes indicating a distinct interferon-associated phenotype in these surviving cells. To confirm this, we analyzed a panel of interferon-related genes and identified a similar expression pattern to *STAT1* in this subgroup (Figure E2A-F in the Online Data Supplement). Specifically, antiviral genes *IFITM2* and *IFITM3* were upregulated in the IPAH trajectory subgroup that matures into non-classical monocytes as are the *IFNGR3* (interferon gamma receptor 3) and *IFIT2* and *IFIT3* which are key components of IFN signaling.

### Classical monocytes in IPAH show increased susceptibility to cell death

**Fig. 4:**
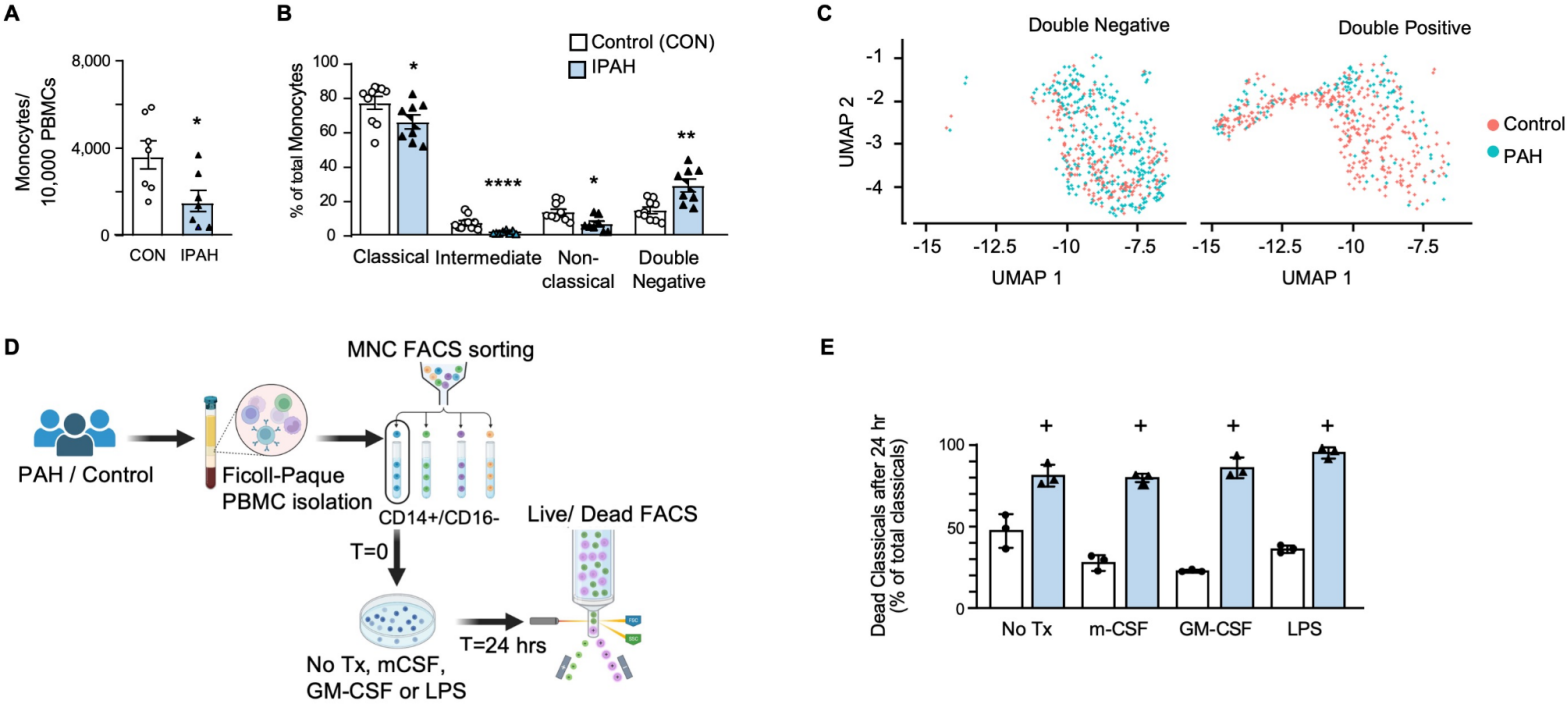
Classical IPAH monocytes are apoptotic. PBMC were isolated from whole blood of Controls and IPAH patients and assessed and/or sorted via FACS. **(A)** Total monocytes per 10,000 PBMC for Control or IPAH (n = 7 each). **(B)** Proportion of monocyte subpopulations of the total monocytes. n = 10 Controls and n = 8 IPAH. (**C**) UMAP of monocyte clusters showing CD14^−^/CD16^−^ cells, and CD14^+^/CD16^+^ cells for Control and IPAH. **(D)** Schematic outlining the approach to assess the fate of IPAH classical monocytes in vitro over 24 h. Whole blood from Controls (n = 3) and IPAH (n = 3) was FACS sorted to collect CD14^+^/CD16^−^ classical monocytes at T = 0. Classical monocytes were cultured for 24 h with or without stimulation by mCSF, GM-CSF or LPS, and viability was assessed via FACS with a Zombie NIR live/dead stain. **(E)**. Dead classical monocytes following 24 h of culture with or without stimulating factors. n = 3 biological replicates. Bars represent mean ± SEM. *p < 0.05, **p < 0.01, ***p < 0.001 by Mann-Whitney non-parametric t-test (A, B & E). For panel E, n = 3 biological replicates were used. The ‘+’ symbol indicates that the minimum achievable *P*-value was reached using a non-parametric t-test with *n* = 3

Based upon the trajectory analysis, we investigated the propensity of CD14^high^ classical IPAH monocytes for cell death. IPAH patients exhibited fewer circulating total monocytes (Fig. 4A). To confirm whether this reduction was specifically due to a decrease in CD14^high^ subpopulations, we conducted additional analysis on samples from the same individuals as Fig. 4A and included additional control and IPAH subjects, examining classical, intermediate, and non-classical monocytes (Fig. 4B). Interestingly, a fourth population characterized as CD14^−^ and CD16^−^ (double negative), was expanded in the IPAH monocytes (Fig. 4B). This finding was further validated in the scRNAseq dataset, which showed an increased presence of this double-negative population in the IPAH compared to the control group (Fig. 4C).

To characterize this expanded IPAH double negative monocyte population and investigate whether the reduction in IPAH monocytes is due to classical monocyte cell death we isolated classical monocytes from PBMC at baseline (T = 0) using FACS sorting (Figure E3A in the Online Data Supplement). This T = 0 population exhibited slight heterogeneity in size compared to controls (Figure E3B in the Online Data Supplement). After 24 h of culture in standard monocyte media without stimulating factors (Fig. 4D), FACS analysis revealed two distinct populations in both the IPAH and control groups (Figure E3C) in the Online Data Supplement). The smaller monocytes were CD14^−^ and CD16^−^ (double negative), while the larger cells were either CD14^+^ classical or CD14^+^/CD16^+^ intermediate monocytes (Figure E3C in the Online Data Supplement). A live/dead stain (gating shown in Figure E3D in the Online Data Supplement) confirmed that the smaller population was in the preliminary stages of cell death. After 24 h in culture, 81.2% of IPAH classical monocytes underwent spontaneous apoptosis compared to 52.7% of control classical monocytes (Fig. 4E).

Spontaneous cell death of monocytes in vitro is a well-documented phenomenon [13, 38] and is characterized by the loss of CD14 from the monocyte cell surface [39, 40]. This natural process of cell turnover can be mitigated when monocytes are stimulated with cytokines and/or toxins either in vitro or in vivo [38]. Upon stimulating the T = 0 classical monocytes with macrophage colony stimulating factor (m-CSF), granulocyte macrophage colony stimulating factor (GM-CSF) or lipopolysaccharide (LPS), there was decreased cell death in the control but not in the IPAH monocytes (Fig. 4E). The observed cell death may reflect apoptosis or alternative pathways, such as necrosis, which could be further investigated using additional markers or functional assays.

### Decreased BMPR2 is linked to reduced CD14 expression

**Fig. 5:**
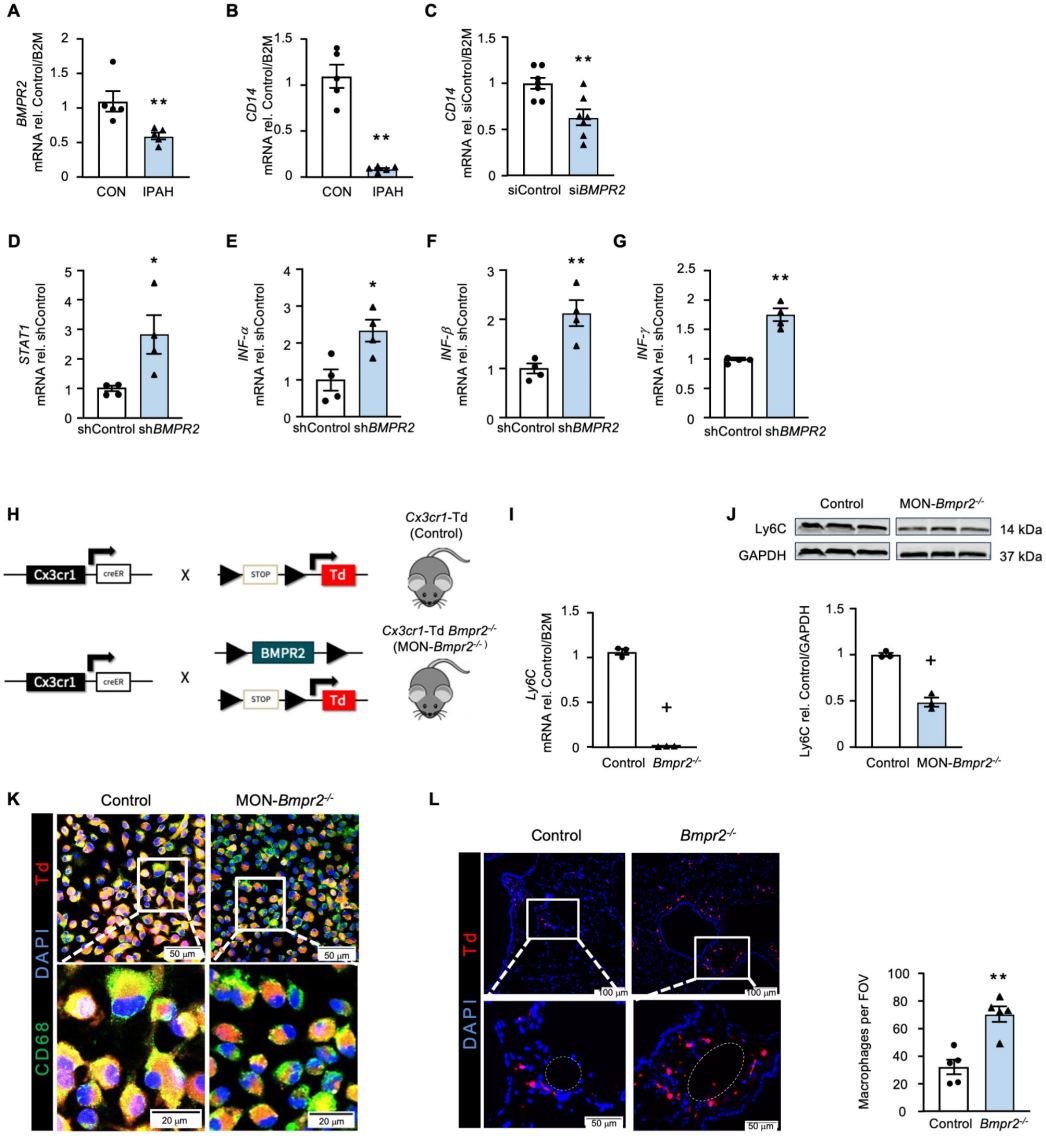
Decreased BMPR2 is linked to decreased CD14 expression. **(A)** RT-qPCR of *BMPR2* gene expression in classical monocytes of IPAH patient. **(B)** RT-qPCR of *CD14* gene expression in classical monocytes of IPAH patients. **(C)** RT-qPCR of *CD14* gene expression in THP1 monocyte cell line, where *BMPR2* was decreased via treatment with siRNA targeting *BMPR2* (si*BMPR2*) vs. non-targeting siRNA (siControl). **(D-G)** RT-qPCR of *STAT1*,* IFN-α*,* IFN-β and IFN-γ* gene expression in THP1 monocyte cell line, where *BMPR2* was decreased via treatment with shRNA targeting *BMPR2* (sh*BMPR2*) vs. non-targeting shRNA (shControl) **(H)** Schematic outlining the breeding strategy used to generate mice with tomato (Td) fluorescent monocyte-specific tag and a conditional, monocyte specific *Bmpr2* deletion (MON-*Bmpr2*^*−/−*^, *Cx3cr1*Td *Bmpr2*^*−/−*^), and Control mice (Control; *Cx3cr1Td*). Details are provided in the Methods. **(I)** RT-qPCR of *Ly6c* (mouse *CD14*) gene expression in circulating monocytes of the MON-*Bmpr2*^*−/−*^ mouse, compared to Control. *n* = 3 groups, each consisting of pooled blood from 3 mice. **(J)** LY6C protein in MON-*Bmpr2*^*−/−*^ bone marrow derived macrophages (mBMDM) assessed via western blot, with representative blot image. **(K)** Representative microscopic images of mBMDM stained with CD68 (green), DAPI (blue) and tamoxifen induced Td (red). MON-*Bmpr2*^*−/−*^ mBMDM were consistently smaller compared to Control. Scale bar = 50 μm, 20 μm (zoom). **(L)** Representative microscopy images with small pulmonary artery (white dashed outline) zoom insert, of lung sections of mice, labeled with DAPI (blue) and showing Td (red) labelled macrophages following hypoxia. Scale bar = 100 μm, 50 μm (zoom). With quantification of macrophages per field of view (FOV). Bars represent mean ± SEM. *p < 0.05, **p < 0.01, by Mann-Whitney non-parametric t-test (A, B, J). For E, F, H, I, *n* = 3 biological replicates; + denotes the minimum achievable *P*-value for *n* = 3 was reached by the non-parametric t test

We next investigated whether the decreased *CD14* mRNA expression in IPAH classical and intermediate monocytes may be linked to reduced *BMPR2* mRNA expression and/or BMPR2 function observed in patients with PAH, including those without a *BMPR2* genetic mutation [3, 41]. Both *BMPR2* and *CD14* mRNA levels were decreased in IPAH classical monocytes (Fig. 5A-B). When we reduced *BMPR2* in THP1 cells (Figure E4A in the Online Data Supplement), *CD14* gene expression was also decreased (Fig. 5C). This decrease in *BMPR2* and *CD14* in THP1 resulted in increased *STAT1*,* IFNα*,* IFNβ*, *IFNγ* (Fig. 5D-G) consistent with the scRNAseq transcriptome of the IPAH monocytes and reflecting a STAT1-IFN response.

Consistent with human cells, circulating monocytes from a monocyte-specific conditional *Bmpr2* knockout mouse, *Cx3cr1*Cre inducible Td Tomato- *Bmpr2*^*−/−*^ (MON-*Bmpr2*^*−/−*^) (Fig. 5H and E4B in the Online Data Supplement), showed decreased *Ly6c* (mouse CD14) mRNA and protein compared to *Cx3cr1*- inducible Td tomato without floxed *Bmpr2* (control) mice (Fig. 5I, J**)**. Using this Cre- inducible system, we isolated mouse bone marrow derived macrophages (mBMDM) from MON-*Bmpr2*^*−/−*−^ mice (Figure E4C in the Online Data Supplement) and cultured them with Tamoxifen to induce Cre recombination of the Td Tomato fluorescence protein. Co-localization of Td Tomato (red) and CD68 (green) confirmed successful *Bmpr2* knockdown (Fig. 5K). We then exposed MON*-Bmpr2*^*−/−*^ and littermate control mice to 3 weeks of hypoxia (10% O_2_). As in previous studies [42], hypoxia was not sufficient to cause more severe or persistent pulmonary hypertension in MON-*Bmpr2*^*−/−*^ male or female mice (Figure E5D-F in the Online Data Supplement), but there was increased recruitment of circulating monocytes into the pulmonary arterial perivascular space in MON*-Bmpr2*^*−/−*^ vs. littermate control mice (Fig. 5L). A previous study using MON-*Bmpr2* knockout mice induced by *LysM-Cre* found that the VEGF receptor inhibitor Sugen 5416, when combined with hypoxia was required to induce more severe pulmonary hypertension than in control mice [6, 9].

### Functional studies related to loss of *BMPR2* in human monocytes

To pursue the link between loss of BMPR2 and CD14 in human monocytes and to carry out functional studies, we used iPSC-derived monocytes (iMono) from three HPAH patients harboring a loss of function *BMPR2* mutation, and from three age and gender matched healthy controls. iMono were differentiated in parallel under the same conditions and validated with the myeloid lineage markers CD64, CD45 and CD11b (Figure E5A and E5B in the Online Data Supplement).

**Fig. 6:**
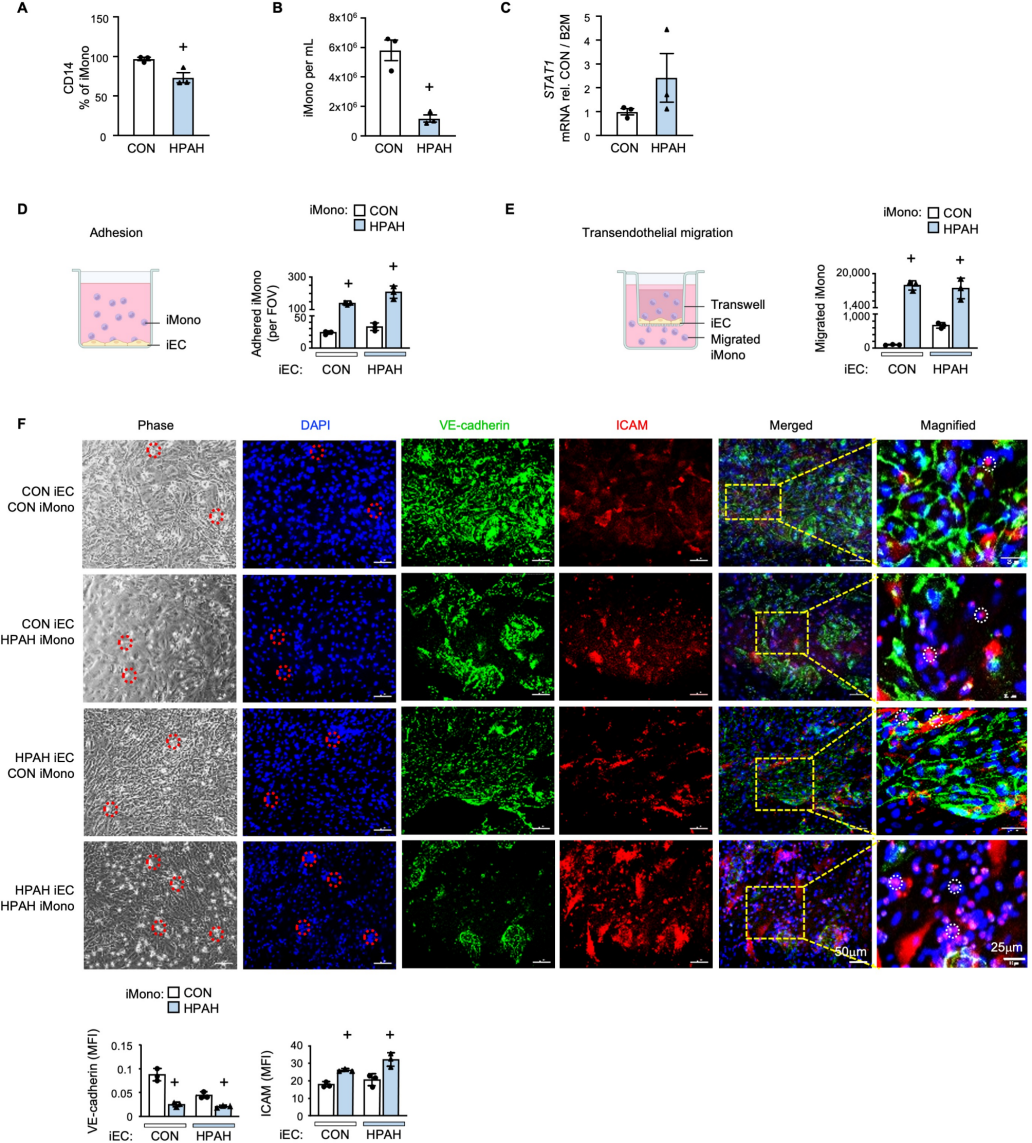
Functional studies related to loss of *BMPR2* in human monocytes. Induced pluripotent stem cells (iPSC) derived from HPAH patients or Controls were differentiated into induced monocytes (iMono) using a stepwise differentiation protocol outlined in the Methods. iMono of HPAH patients and Controls were compared for **(A)** CD14 cell surface expression, assessed by FACS; **(B)** Number of iMono per mL. on day 19 of differentiation. **(C)***STAT1* gene expression by RT-qPCR. **(D)** Schematic demonstrating iEC and iMono co-culture approach for assessing adhesion. iMono adhesion to Control or HPAH iEC, per field of view. **(E)** Schematic demonstrating iEC and iMono co-culture approach for assessing transendothelial migration. Migrated of iMono across Control or HPAH iEC. **(F)** VE-cadherin and ICAM1 expression in co-culture of iMono and iEC with quantification based on mean fluorescent intensity (MFI) of 10 FOV for each replicate. Representative images of the iEC/iMono co-culture including phase, DAPI (blue) VE-cadherin (CDH5) (green) and ICAM1 (red). Red dotted outline on phase and DAPI images show adhered iMono. White dotted outline on merged images show corresponding iMono (pink). Bars represent mean ± SEM. For all experiments, *n* = 3 biological replications. + denotes the minimum achievable *P*-value for *n* = 3 was reached by the non-parametric t test

iMono derived from *BMPR2* mutant HPAH iPSC had decreased CD14 cell surface expression compared to controls (Fig. 6A), and impaired differentiation efficiency, shown by reduced cell yield (Fig. 6B), as well as increased *STAT1* (Fig. 6C).

Functional co-culture studies were conducted with iMono and iPSC induced-endothelial cells (iEC) differentiated from the same iPSC lines (Fig. 6D-F) and validated by acetylated LDL uptake (Figure E5C in the Online Data Supplement). HPAH vs. control iMono exhibited a marked increase in adhesion (Fig. 6D) and transendothelial migration (Fig. 6E) with both control and HPAH iEC monolayers. A smaller increase in transendothelial migration was observed with control iMono on HPAH iEC compared to control iEC, as previously shown with neutrophils [22]. Increased intercellular adhesion molecule (ICAM1) was observed in HPAH iMono compared to control iMono (Fig. 6F), consistent with studies indicating that *Bmpr2* knockout monocyte lineage mice (*Bmpr2*^*KO*^) show elevated soluble ICAM1 in the circulation and in lung tissue following Sugen/hypoxia-induced pulmonary hypertension [6]. The observed increase in ICAM1 (Fig. 6F) may explain the increased adhesion and the reduction in VE-cadherin (Fig. 6F) may explain the increased transendothelial migration.

## Discussion

Most studies investigating myeloid lineage cells in PAH focused on macrophages and their role in the obliteration of the distal pulmonary arteries [6, 43–46]. Florentin et al. showed that pathogenic macrophages in pulmonary hypertension rodent models originate from the recruitment and infiltration of circulating monocytes into the perivascular space of pulmonary arteries [9]. Recently, an increase in pro-inflammatory CCR2^+^ macrophages in the right ventricle in a rat PH model, with high expression of the NLRP3 inflammasome pathway was reported [47]. Treatment with a NLRP3 inhibitor (MCC950) reduced NLRP3 activation and regression of pulmonary vascular disease. Despite these findings and studies of monocytes in other vascular diseases such as atherosclerosis [48], systemic lupus erythematosus [49], peripheral artery occlusive disease and coronary artery disease [50], the characteristics of monocytes that contribute to PAH mediated pulmonary artery remodeling are relatively unknown.

We show that circulating classical IPAH monocytes exhibit an aberrant maturation trajectory, with premature termination attributed to decreased *CD14* and related downstream immune response activation genes *FOS*,* JUNB*,* DUPSP1 and ANXA1*. These findings are consistent with cell death triggered by loss of CD14 [39, 40]. Classical IPAH monocytes that are able to mature to intermediate and non-classical monocytes show persistent expression of elevated *STAT1* and interferon genes indicative of an interferonopathy [51]. Previous studies have linked PAH vascular remodeling to upregulated type I IFN signaling [52], and blood genome meta-analysis show IFN as one of the top transcriptional pathways in PAH [53]. Elevated *STAT1* has been related to heightened adhesion, invasiveness, and preferential differentiation into an M1 inflammatory macrophage [54, 55]. Other genes increased in expression in PAH monocytes such as *PPDPF* have not previously been studied in monocytes, but could be further investigated as an inhibitor of mTOR [56] and a pro-apoptotic factor [57]. Induction of HLA-B could be evaluated in response to the high HERV-K levels in PAH monocytes as HLA-B is increased in response to viral infections [58].

In vascular diseases, IFN-mediated inflammation promotes the expression of vascular adhesion molecules such as ICAM1, inhibits vascular endothelial growth factor (VEGF) [59], and reduces VE-cadherin, increasing vascular permeability [60]. Through IFN activation, STAT1 can specifically promote *ICAM1* gene expression [61]. Here we demonstrate that co-culture of HPAH iMono with either control or HPAH iEC results in increased ICAM1 mRNA in iMono, leading to an adhesive and invasive phenotype capable of penetrating the endothelial barrier and invading the perivascular space related to loss of VE-cadherin [54, 61, 62].

Our studies link the aberrant transcriptional and maturation profile of PAH monocytes to reduced *BMPR2* expression seen in all forms of PAH [3]. The bone morphogenetic protein pathway drives myeloid cell maturation [63] indicating that suppression of this pathway could result in aberrant recruitment of monocytes and differentiation to macrophages with a decreased ability to resolve inflammation [44]. Elevated monocyte ICAM1 has been linked to inflammation and vascular injury resulting from myocardial infarction [64, 65], and elevated ICAM1 in the *BMPR2* mutant PAH monocyte had a much greater impact on enhanced adhesion and transendothelial migration than PAH EC.

A limitation of the mouse model presented is that deletion of *Bmpr2* in circulating monocytes did not result in worse hypoxia-induced PH. However, previous studies using a similar model reported that the addition of the VEGF inhibitor was necessary to show the more severe PH phenotype [6]. It is possible that infectious/inflammatory stimuli such as Schistosoma exposure that induces CCL2 mediated CCR4^+^ monocyte recruitment [66] or γ-herpesvirus 68 [67] may also bring out the more severe PH phenotype in mice with loss of *Bmpr2* in monocytes.

Monocyte derivatives control tissue microenvironments in tumors and in chronic inflammatory diseases [68–70] and inhibitors of monocyte signaling have been applied in cancer [70] and in PAH where STAT1 inhibition is effective in preventing PAH [71]. Thus, emerging PAH therapies should be evaluated for their impact in reversing the aberrant monocyte phenotype.

## Electronic supplementary material

Below is the link to the electronic supplementary material.

Supplementary Material 1

Supplementary Material 2

## Data Availability

Data availability: The Single cell RNA seq datasets supporting the conclusions of this article are available in the in the NCBI Gene Expressing OmEtnibus (GEO) with accession number: GSE233189. [https://ncbi.nlm.nih.gov/geo/query/acc.cgi?acc=GSE233189].

## Code availability

For the single cell RNA seq, the raw sequencing files and aggregated matrix can be downloaded at Gene Expression Omnibus under GEO number GSE233189. The analysis code can be found at https://github.com/xzhou7/scRNA_PBMC.

## Abbreviations

PAH: Pulmonary arterial hypertension
BMPR2: Bone morphogenetic protein receptor type 2
HPAH: Hereditary pulmonary arterial hypertension
IPAH: Idiopathic pulmonary arterial hypertension
PH: Pulmonary hypertension
scRNAseq: Single cell RNA sequencing
STAT1: Signal transducer and activator of transcription 1
FOS: Fos proto-oncogene
JUNB: JunB proto-oncogene
iPSC: Induced pluripotent stem cells
iMONO: Induced monocytes
PBMC: Peripheral blood mononuclear cells
PAEC: Pulmonary arterial endothelial cells
EC: Endothelial cells
iEC: Induced endothelial cells
PCR: Polymerase chain reaction
LDL: Low density lipoprotein
FBS: Fetal bovine serum
DC: Dendritic cells
NK: Natural killer
DE: Differentially expressed
IFN: Interferon
PPDPF: Pancreatic progenitor cell differentiation and proliferation factor
FTL: Ferritin light chain
MTRNR2L8: MT-RNR2 Like 8
TSC22D3: TSC22 domain family member 3
DUSP1: Dual specificity phosphatase 1
PLP2: Proteolipid protein 2
ANXA10: Annexin A10
IFITM3: Interferon induced transmembrane protein 3
m: CSF-macrophage colony stimulating factor
GM: CSF-granulocyte macrophage colony stimulating factor
ICAM: Intercellular adhesion molecule 1
VEGF: Vascular endothelial growth factor
