# Supplementary material for: Altered maturation and activation state of circulating monocytes is associated with their enhanced recruitment in pulmonary arterial hypertension
Source: Respir Res. 2025 Apr 15;26:148. doi: 10.1186/s12931-025-03182-0 (PMC11998417; doi:10.1186/s12931-025-03182-0)

<u>Uncropped blots:</u> Harper, Zhou *et al.*, “Altered Maturation and Activation State of Circulating Monocytes Is Associated with Their Enhanced Recruitment In Pulmonary Arterial Hypertension.”

NOTES:

- Samples were loaded and ran on 2 gels in parallel
- Precision Plus protein dual color standards were used for all of the MW markers

Figure 5J:

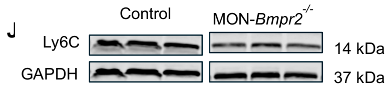

Ly6C

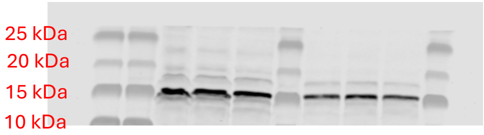

GAPDH

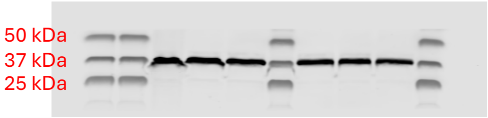

Supplement: Supplementary file 1 — Supplementary Material 1 [file 12931_2025_3182_MOESM1_ESM.pdf]
