# Supplementary material for: Altered maturation and activation state of circulating monocytes is associated with their enhanced recruitment in pulmonary arterial hypertension
Source: Respir Res. 2025 Apr 15;26:148. doi: 10.1186/s12931-025-03182-0 (PMC11998417; doi:10.1186/s12931-025-03182-0)
Supplement: Supplementary file 2 — Supplementary Material 2 [file 12931_2025_3182_MOESM2_ESM.pdf]

# Online Data Supplement

*Rebecca L. Harper, *Xin Zhou, David P. Marciano, Aiqin Cao,

Lingli Wang, Guibin Chen, Mir S. Adil, Wenyu Zhou, Peter Maguire,

Shanthi Deivanayagam, Quan Yu, Vignesh Viswanath, Dan Yang, Marcy Martin,

Sarasa Isobe, Shoichiro Otsuki, Jordan Burgess, Audrey Inglis, Devon Kelley,

Patricia A. del Rosario, Andrew Hsi, Francois Haddad, Roham T. Zamanian,

Manfred Boehm, Michael P. Snyder, #Marlene Rabinovitch

* These authors contributed equally to this work

# Corresponding Author: marlener@stanford.edu

## SUPPLEMENTARY TABLES

### Table E1: Demographics and assays used for PAH patients

| ID | Age (yr) | Gender | Race | Ethnicity | Diagnosis (Mutation) | mPAP (mmHg) | PVR (WU) | 6MWD (m) | Medications | NYHA Class | Assay |
|---|---|---|---|---|---|---|---|---|---|---|---|
| PAH1 | 20 | F | Caucasian | Non-Hispanic | IPAH | 33 | 6.84 | 675 | Ambrisentan, tadalifil | 1/2 | FACS |
| PAH2 | 28 | F | Caucasian | Non-Hispanic | IPAH | 51 | 12.5 | 512 | Flolan | 1/2 | FACS |
| PAH3 | 55 | F | Caucasian | Hispanic or Latino | IPAH | 62 | 16.5 | 366 | Flolan, ambrisentan, | 2 | FACS |
| PAH4 | 53 | M | Caucasian | Hispanic or Latino | IPAH | 53 | 5.3 | 293 | Macitentan, sildenafil | 3 | FACS |
| PAH5 | 54 | F | Caucasian | Non-Hispanic | IPAH | 48 | 13.5 | 220 | Remodulin, sildenafil | 2 | FACS |
| PAH6 | 63 | F | Caucasian | Non-Hispanic | IPAH | 44 | 8.2 | 504 | Bosentan, sildenafil | 3 | FACS |
| PAH7 | 66 | F | Caucasian | Non-Hispanic | IPAH | 35 | 4.08 | 594 | Macitentan, sildenafil | NA | FACS |
| PAH8 | 57 | F | Caucasian | Non-Hispanic | IPAH | 36 | 7.62 | 408 | Veletri | 1 | FACS scRNAseq |

| ID | Age (yr) | Gender | Race | Ethnicity | Diagnosis (Mutation) | mPAP (mmHg) | PVR (WU) | 6MWD (m) | Medications | NYHA Class | Assay |
|---|---|---|---|---|---|---|---|---|---|---|---|
| PAH9 | 41 | F | Caucasian | Non-Hispanic | IPAH | 32 | 5.15 | NA | Macitentan, sildenafil | 2 | FACS |
| PAH10 | 51 | F | Asian | Non-Hispanic | IPAH | 58 | 9.7 | 562 | Epoprostenol, tadalafil | 2 | FACS |
| PAH11 | 59 | F | Caucasian | Non-Hispanic | IPAH | 50 | 5.5 | 421 | Ambrisentan, sildenafil, epoprostenol | 2 | scRNAseq |
| PAH12 | 56 | F | Caucasian | Non-Hispanic | IPAH | 39 | 5.36 | 686 | Ambrisentan, sildenafil | 1/2 | scRNAseq |
| PAH13 | 66 | F | Caucasian | Hispanic or Latino | IPAH | 52 | 8.78 | 466 | Ambrisentan, sildenafil | 3 | scRNAseq |
| PAH14 | 45 | F | Asian | Non-Hispanic | IPAH | 65 | 16.66 | 504 | Veletri, sildenafil | 2 | scRNAseq |
| PAH15 | 52 | F | Caucasian | Non-Hispanic | IPAH | 48 | 4.96 | 594 | Macitentan, sildenafil | 2 | scRNA-seq |
| PAH16 | 33 | F | African American | Non-Hispanic | HPAH (c.1471C>T p.R491W) | 48 | 15.57 | 326.1 | IV epoprostenol, bosentan, sildenafil, inhaled treprostinil | NA | iMono/iMac |
| PAH17 | 37 | M | Caucasian | Non-Hispanic | HPAH (c.1471C>T p.R491W) | 77 | 14.22 | 309 | sildenafil, sitaxsentan, ambrisentan, IV epoprostenol, Imatinib (investigational medication), IV treprostinil | NA | iMono/iMac |

| ID | Age (yr) | Gender | Race | Ethnicity | Diagnosis (Mutation) | mPAP (mmHg) | PVR (WU) | 6MWD (m) | Medications | NYHA Class | Assay |
|---|---|---|---|---|---|---|---|---|---|---|---|
| PAH18 | 57 | M | Caucasian | Non-Hispanic | HPAH (c.354T>G p.C118W (BMPR2) | 61 | 11.3 | 420 | None | 1 | iMono/iMac |

[1] Diagnosis: IPAH, Idiopathic PAH; D&T Drug and toxin associated PAH, COPD, Chronic obstructive pulmonary disease

[2] PAP: Mean Pulmonary Arterial Pressure, test closest to the time of blood draw

[3] PVR: Pulmonary vascular resistance in Woods Units, test closest to the time of blood draw

[4] 6MWD: Distance (in meters) walked in six minutes; test closest to the time of blood draw

[5] NYHA: New York Heart Association functional classification

NA, data not available

**Table E2: Demographics of Donor Controls, and Assays**
*Samples obtained at Stanford Blood Center. All other CNTL samples were obtained from Stanford Biobank

| ID | Age | Gender | Race/ Ethnicity | Ethnicity | Assay |
|---|---|---|---|---|---|
| CNTL1* | 30 | M | Caucasian | Non-Hispanic | FACS |
| CNTL2* | 79 | M | Caucasian | Non-Hispanic | FACS |
| CNTL3* | 51 | M | Caucasian | Hispanic or Latino | FACS |
| CNTL4 | 47 | F | Caucasian | Non-Hispanic | scRNAseq<br>FACS |
| CNTL5 | 50 | F | Asian | Non-Hispanic | scRNAseq<br>FACS |
| CNTL6 | 37 | F | Asian | Non-Hispanic | scRNAseq<br>FACS |
| CNTL7 | 34 | F | Caucasian | Hispanic or Latino | scRNAseq<br>FACS |
| CNTL8* | 63 | F | Caucasian | Non-Hispanic | FACS |
| CNTL9* | 72- | M | Caucasian | Non-Hispanic | FACS |
| CNTL10* | 76 | M | Caucasian | Non-Hispanic | FACS |
| CNTL11 | 35 | F | Caucasian | Non-Hispanic | scRNAseq |
| CNTL12 | 36 | F | Caucasian | Non-Hispanic | scRNAseq |
| CNTL13 | 25 | M | Caucasian | Non-Hispanic | iMono/iMac |
| CNTL14 | 46 | F | Caucasian | Non-Hispanic | iMono/iMac |
| CNTL15 | 49 | M | Caucasian | Non-Hispanic | iMono/iMac |

**Table E3: Reagents and Resources**

| REAGENT or RESOURCE | SOURCE | IDENTIFIER/SEQUENCE |
|---|---|---|
| **Antibodies** | | |
| Anti-human ICAM1 | Abcam | Cat#: ab109361 |
| Anti-human VE-Cadherin | Santa Cruz | Cat#: sc-9989 |
| Anti-CD68 | Abcam | Cat#: ab201340 |
| Anti-GAPDH | Cell Signaling | Cat#: 2118 |
| Anti-Ly6C | eBioscience | Cat#:14-5931-85 |
| Alexa Fluor488 donkey anti mouse IgG (H +L) | Invitrogen | Cat#: A21202 |
| Alexa Fluor594 goat anti rabbit IgG (H +L) | Invitrogen | Cat#: A11012 |
| **siRNA** | | |
| SMARTpool ON-TARGETplus- BMPR2 | Origene | Cat#: L-001230-00-005 |
| SMARTpool ON-TARGETplus- Control | Origene | Cat#: D-001230-01 |
| **Reagents, Cells, Chemicals, Peptides, and Recombinant Proteins** | | |
| THP1 Cells | ATCC | Cat#: TIB-202 |
| HEK293T | ATCC | Cat#: CRL-3216 |
| shRNA (scramble) | Vector Builder | Cat#: VB010000-0009mxc |
| shRNA (BMPR2) | Vector Builder | Cat#: VB900034-4780ezd |
| DMEM | Gibco | Cat#: 11965092 |
| Polybrene | Santa Cruz | Cat# CAS 28728-55-4 |
| TransIT-TKO™ reagent | Mirus | Cat#: MIR2150 |
| RPMI 1640 | Gibco | Cat#: 11875093 |
| RPMI 1640 | R & D Systems | Cat#: S11150H |
| Monocyte attachment medium | PromoCell | Cat#: C28051 |
| EMEM | Sigma Aldrich | Cat#: M4655 |
| Penicillin and Streptomycin | Invitrogen | Cat#: 15140122 |

| REAGENT or RESOURCE | SOURCE | IDENTIFIER/SEQUENCE |
|---|---|---|
| Penicillin and Streptomycin | Gibco | Cat#: 15070063 |
| ProLong™ Gold Antifade Mountant with DAPI (Invitrogen, Cat#: P36931) | Invitrogen | Cat#: P36931 |
| DAPI | ThermoFisher | Cat#: D1306 |
| Fibronectin from human plasma | Corning | Cat#: 54008 |
| Ficoll-Paque (Histopaque) | Sigma Aldrich | Cat#: 10771 |
| PBS | Invitrogen | Cat#: 10010023 |
| UltraPure 0.5M EDTA, pH 8.0 | EMD Millipore | Cat#: 15575020 |
| 1M Tris-HCL | Life Technologies | Cat#: 15567-027 |
| UltraPure 10%SDS | Life Technologies | Cat#: 24730-020 |
| Bovine Serum Albumin | Sigma Aldrich | Cat#: A3059-500G |
| Tri-Reagent | Sigma Aldrich | Cat#: T9424 |
| Corning® Matrigel® Matrix Growth Factor Reduced Matrigel | Corning | Cat#: 354230 |
| Essential 8 Medium | Gibco | Cat#: A1517001 |
| STEMdiff™ APEL™2 Medium | StemCell Technologies | Cat#: 05275 |
| Rock Inhibitor Y-27623 | Tocris Bioscience | Cat#: 1254 |
| TrypLE Trypsin | Invitrogen | Cat#: 12563 |
| StemPro™-34 SFM | Gibco | Cat#: 10639011 |
| EGM™ 2 Endothelial Cell Growth Medium BulletKit™ | Lonza | Cat#: CC-3162 |
| Collagen I Coated Plates (Corning BioCoat) | Corning | Cat#: 354450 |
| Human recombinant Bone morphogenetic protein 4- rhBMP4 | R&D Systems | Cat#: 314-BP-050 |
| Human recombinant Vascular endothelial growth factor-rhVEGF | Gibco | Cat#: PHC9394 |
| Human recombinant Basic fibroblast growth factor-rhbFGF | Prepotech | Cat#: 100-18B |
| Human recombinant stem cell factor-rhSCF | StemCell Technologies | Cat#: 2830 |

| **REAGENT or RESOURCE** | **SOURCE** | **IDENTIFIER/SEQUENCE** |
|---|---|---|
| Human recombinant Flt-3 Ligand-rhFlt-3 L | StemCell Technologies | Cat#: 2941 |
| Human recombinant Thrombopoietin-rhTPO | StemCell Technologies | Cat#: 02720 |
| Human Granulocyte-Macrophage Colony-Stimulating Factor-rhGM-CSF | Prepotech | Cat#: 300-03 |
| Human recombinant Macrophage Colony-Stimulating Factor-rhM-CSF | Prepotech | Cat#: 300-25 |
| Lipopolysaccharides from Escherichia coli O111:B4 | Sigma Aldreich | L2880 |
| **Commercial Assays and Kits** | | |
| Falcon® Permeable Support for 24-well Plate with 3.0 µm Transparent PET Membrane | Corning | Cat#: 353096 |
| Power SYBR green PCR Master Mix | Applied Biosystems | Cat#: 4387406 |
| DakoArk | Dako | Cat#: K3954 |
| ON-TARGETplus Human VCL siRNA | Dharmacon | Cat#: L-009288-00-0010 10 nmol |
| Dako Liquid DAB + Substrate Chromogen System | Dako | Cat#: K3468 |
| Monocyte Isolation Kit (BM), mouse | Miltenyi | Cat#: 130-100-629 |
| Qubit High Sensitivity double stranded (ds)DNA Assay | ThermoFisher | Cat#: Q32850 |

**Table E4: FACS Antibody Panel**

| PANEL | TARGET | FLUOROCHROME | CLONE | COMPANY | CATALOG N° |
|---|---|---|---|---|---|
| **Primary Monocyte Sorting Panel** | Viable dye | NIR | | BioLegend | 423105 |
| | CD14 | FITC | MφP9 | BD Biosciences | 347493 |
| | CD16 | PE | NKP15 | BD Biosciences | 347617 |
| | CD56 | APC | HCD56 | BioLegend | 318310 |
| | CD19 | APC | HIB19 | BD Biosciences | 555415 |
| | CD3 | APC | HIT3a | BioLegend | 300312 |
| | CD11c | APC | 3.9 | BioLegend | 301620 |
| **iHSCs and monocytes panel** | Viable dye | Violet | | BioLegend | 423102 |
| | CD64 | PE | 10.1 | BioLegend | 983202 |
| | CD45 | APC | H130 | BioLegend | 304012 |
| | CD14 | FITC | GCD14 | BioLegend | 325604 |
| | CD16 | APC | 3G8 | BioLegend | 302012 |
| | CD11b | PE Cy5 | ICRF44 | BioLegend | 301308 |

**Table E5: Primers used for qPCR and genotyping**

| GENE | SOURCE | FORWARD | REVERSE |
|---|---|---|---|
| Human *BMPR2* | Stanford PAN facility | 5’-CTGCGGCTGCTTCGCAGAAT-3’ | 5’-TGGTGTTGTGTCAGGAGGTGG-3’ |
| Human *B2M* | Stanford PAN facility | 5’-TTCTGGCCTGGAGGCTATC-3’ | 5’-TCAGGAAATTTGACTTTCCATTC-3’ |
| Human *CD14* | Stanford PAN facility | 5’-ACGCCAGAACCTTGTGAGC-3’ | 5’-GCATGGATCTCCACCTCTACTG-3’ |
| Human *STAT1* | Stanford PAN facility | 5’-ATCAGGCTCAGTCGGGGAATA-3’ | 5’-TGGTCTCGTGTTCTCTGTTCT-3’ |
| Human *IFNA* | Stanford PAN facility | 5’- GCCTCGCCCTTTGCTTTACT-3’ | 5’- CTGTGGGTCTCAGGGAGATCA-3’ |
| Human *IFNB* | Stanford PAN facility | 5’- ATGACCAACAAGTGTCTCCTCC-3’ | 5’- GGAATCCAAGCAAGTTGTAGCTC-3’ |
| Human *IFNG* | Stanford PAN facility | 5’- TCGGTAACTGACTTGAATGTCCA-3’ | 5’- TCGCTTCCCTGTTTTAGCTGC-3’ |
| Human *CXCR3* | Stanford PAN facility | 5’- ACGAGAGTGACTCGTGCTGTAC-3’ | 5’- GCAGAAAGAGGAGGCTGTAGAG-3’ |
| Mouse *Bmpr2* | Stanford PAN facility | 5’-GTGTTATGGTCTGTGGGAGAAAT-3’ | 5’-AAAGCGGTACGTTCCATTCTG-3’ |
| Mouse *Ly6c* | Stanford PAN facility | 5’-GAAGCAGTATTGGGTCTGCAA-3’ | 5’-TTGGAAGATTCATGGTGTTGACA-3’ |
| Mouse *B2M* | Stanford PAN facility | 5’-GGCCCATCTTGCATTCTAGGG-3’ | 5’-GCAACGGCTCTATATTGAAGTCA-3’ |
| *CX3cr1Cre*<br>(mutant 230 bp, WT 151 bp) | Stanford PAN facility | Mutant forward:<br>5’-GTTAATGACCTGCAGCCAAG-3’<br>Wild type forward:<br>5’-AGCTCACGACTGCCTTCTTC-3’ | Common reverse:<br>5’-ACGCCCAGACTAATGGTGAC-3’ |
| *Bmpr2* deletion<br>(328 bp) | Stanford PAN facility | 5’-CGTCGACCTCGAATAACTTCG TATAGC-3’ | 5’-GAGGCATGCCTCAAACTCCACTG TG-3’ |
| *Bmpr2 floxed*<br>(Floxed 240 bp, WT 170 bp) | Stanford PAN facility | 5’-GGGTCATTCTCATTCAAGCTA CCACG-3’ | 5’-CTTTCAGAAGACCCAAGTCCTGTTC CC-3’ |

| GENE | SOURCE | FORWARD | REVERSE |
|---|---|---|---|
| Td Tomato (Mutant 200 bp) | Stanford PAN facility | 5'-GGCATTAAAGCAGCGTATCC-3' | 5'-CTGTTCCTGTACGGCATGG-3' |
| Mouse *Ly6C* | Stanford PAN facility | 5’-GAAGCAGTATTGGGTCTGCAA-3’ | 5’-TTGGAAGATTCATGGTGTTGACA-3’ |
| Td Tomato (WT 300 bp) | Stanford PAN facility | 5'-AAGGGAGCTGCAGTGGAGTA-3' | 5'-CCGAAAATCTGTGGGAAGTC-3' |

## SUPPLEMENTARY FIGURES

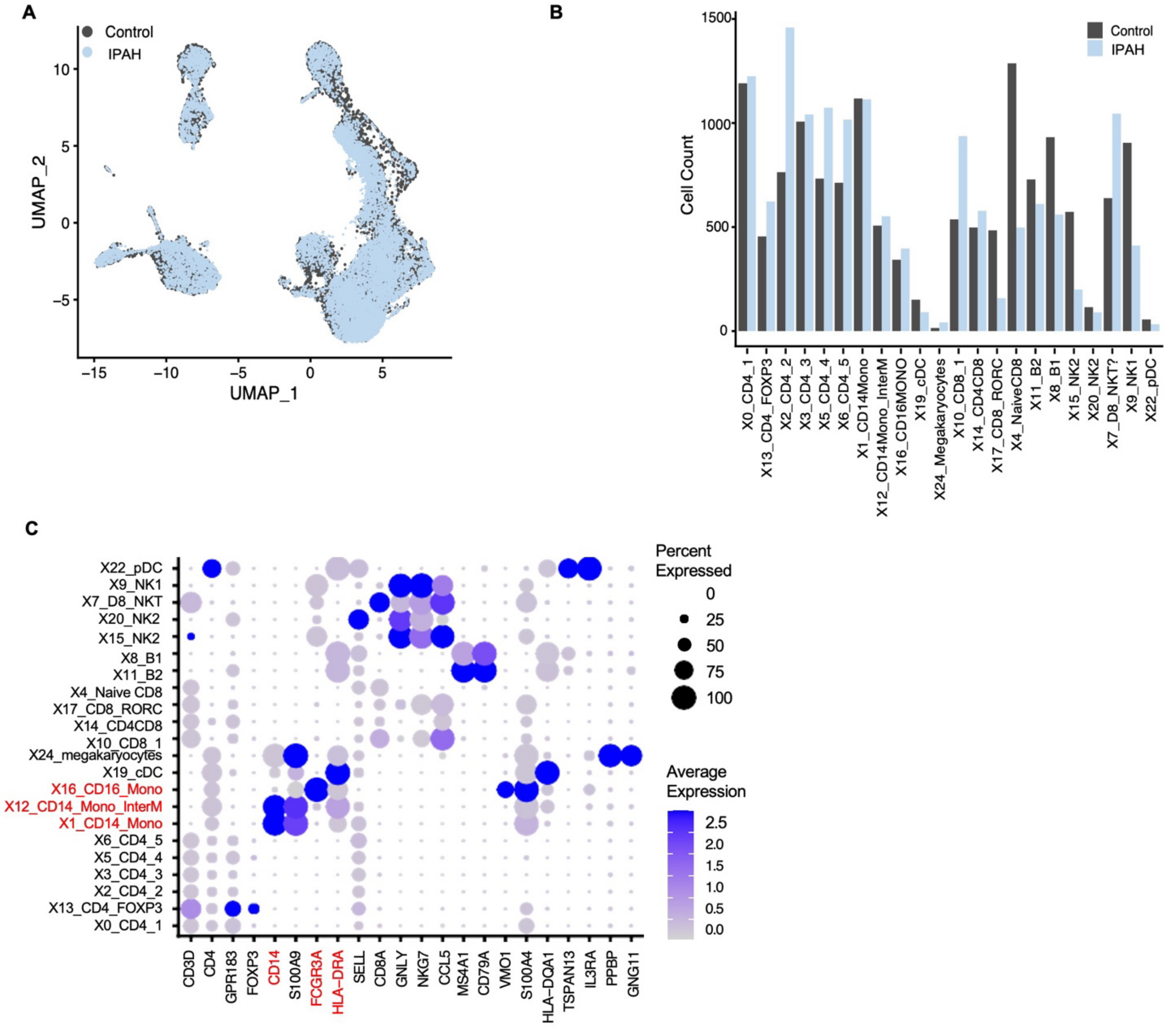


**Figure E1: PBMC cell populations in IPAH and Control Samples**

**(A)** UMAP of donor control and IPAH data integration. Light blue = IPAH, grey = Control. **(B)** Cell count by cluster for both Control and IPAH. **(C)** Dot plot of average expression of combined Control and IPAH (purple color scale) and percentage of expression (dot size) of known cell markers for each cluster with monocyte subpopulations highlighted in red. n=6/group.

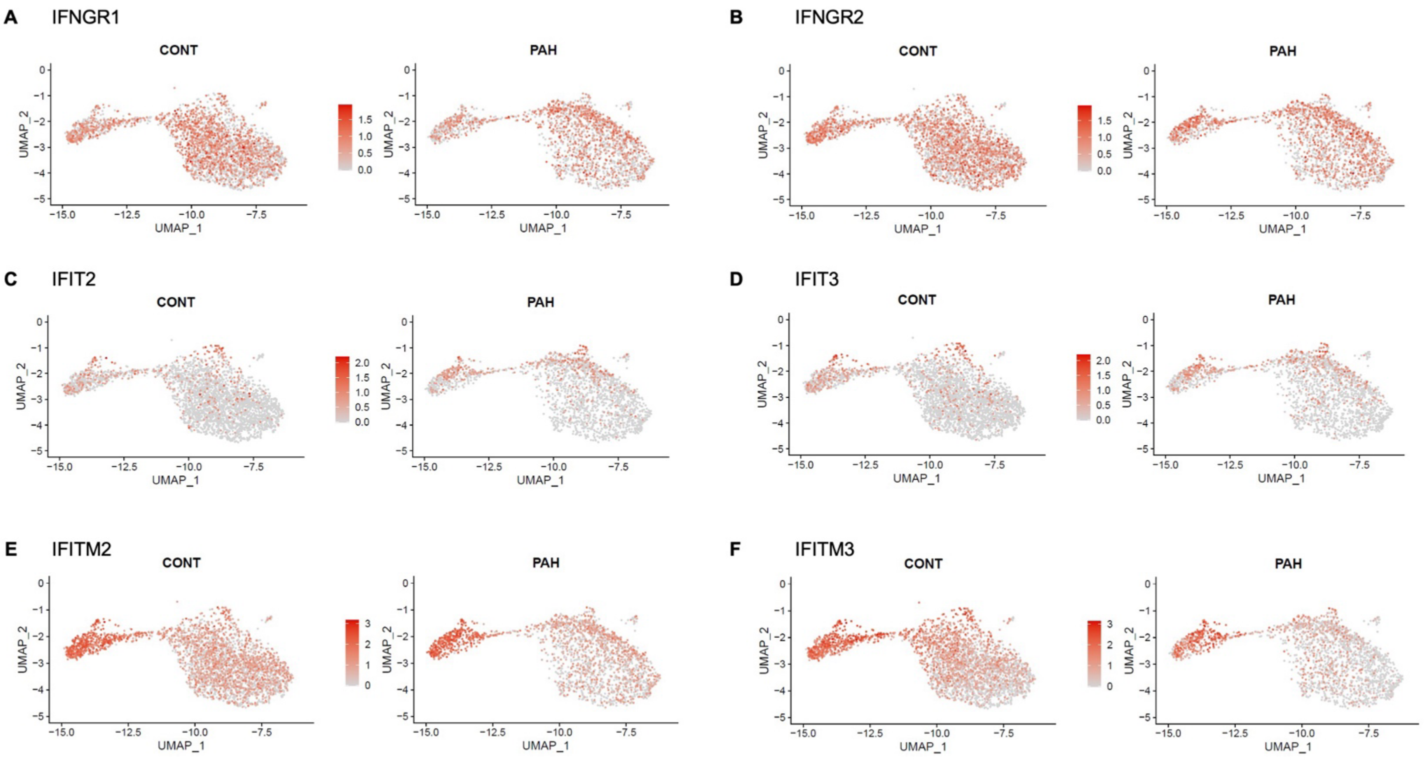


**Figure E2: Trajectory analysis of interferon genes in scRNAseq**

UMAP of monocyte clusters configured for trajectory analysis showing **(A)** IFNGR1, **(B)** IFNGR2, **(C)** IFIT2, **(D)** IFIT3, **(E)** IFITM2, and **(F)** IFITM3 in Control and IPAH. n=6/group.

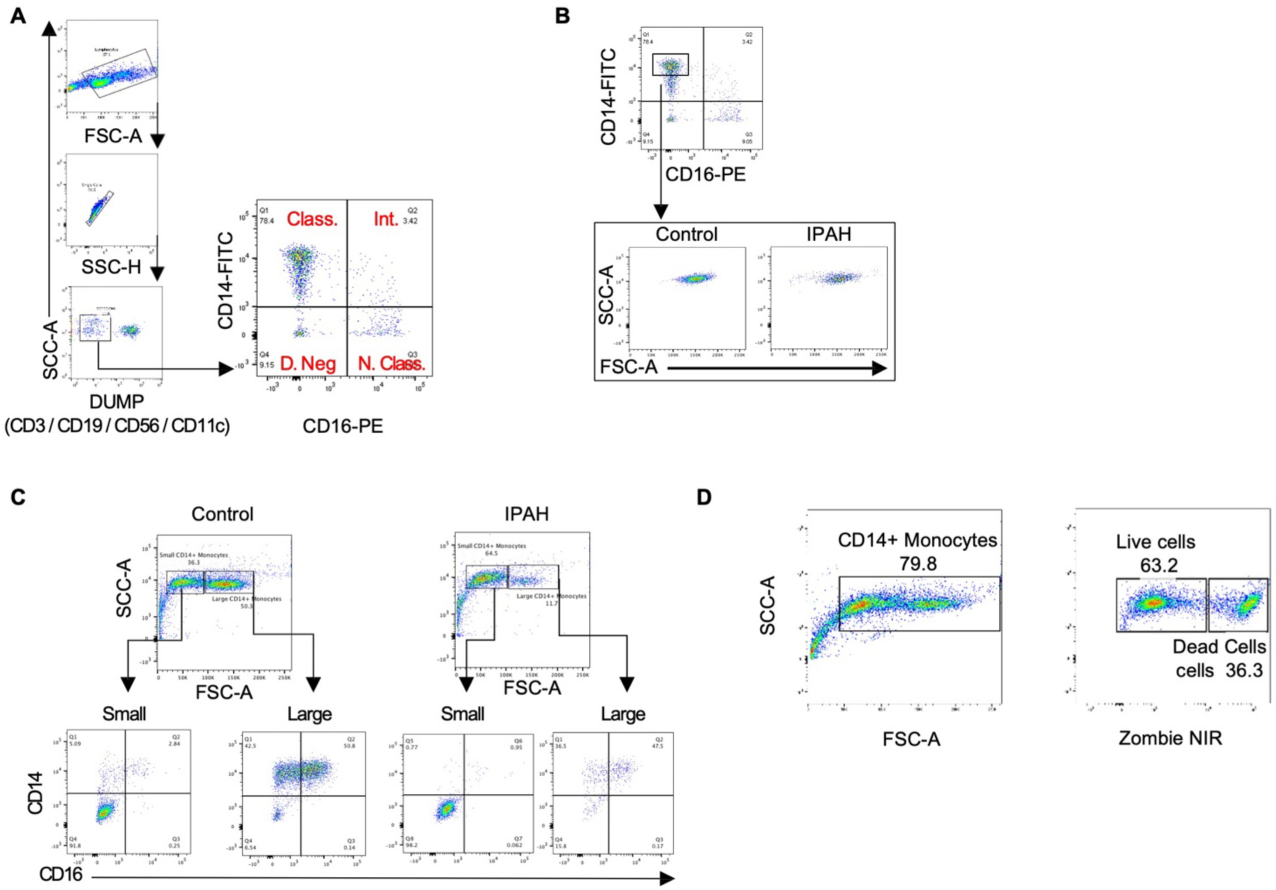


**Figure E3: Representative gating strategy**

**(A)** Representative gating strategy used for identifying, quantifying, and sorting monocyte subpopulations from PBMC to maximize monocyte capture and minimize loss of vulnerable cells. PBMC were stratified based on size and complexity with side-scattered light (SSC-A) and forward-scattered light (FSC-A) to eliminate dead cells and cell debris. The gate is identified as "Lymphocytes". This gated population was filtered to capture only singlets, and from these single cells, all cells that were CD3, CD19, CD56 and CD11c positive were 'dumped', leaving only monocytes for subsequent CD14/ CD16 gating. **(B)** Representative gating by size and complexity of $CD14^+/CD16^-$ classical monocytes at the time of isolation (T=0 hours) shows increased heterogeneity of IPAH classical monocytes compared to control. **(C)** Representative FACS assessment of T=24 hours $CD14^+$ classical monocytes to assess differentiation into intermediate and non-classical subpopulations reveals a bimodal distribution based on size. 'Small' cells are $CD14^-/CD16^-$ and 'Large' cells are either $CD14^+/CD16^-$ classical monocytes, or $CD14^+/CD16^+$ intermediate monocytes. **(D)** Representative FACS live/dead assessment of T=24 hours using Zombie NIR live/dead staining.

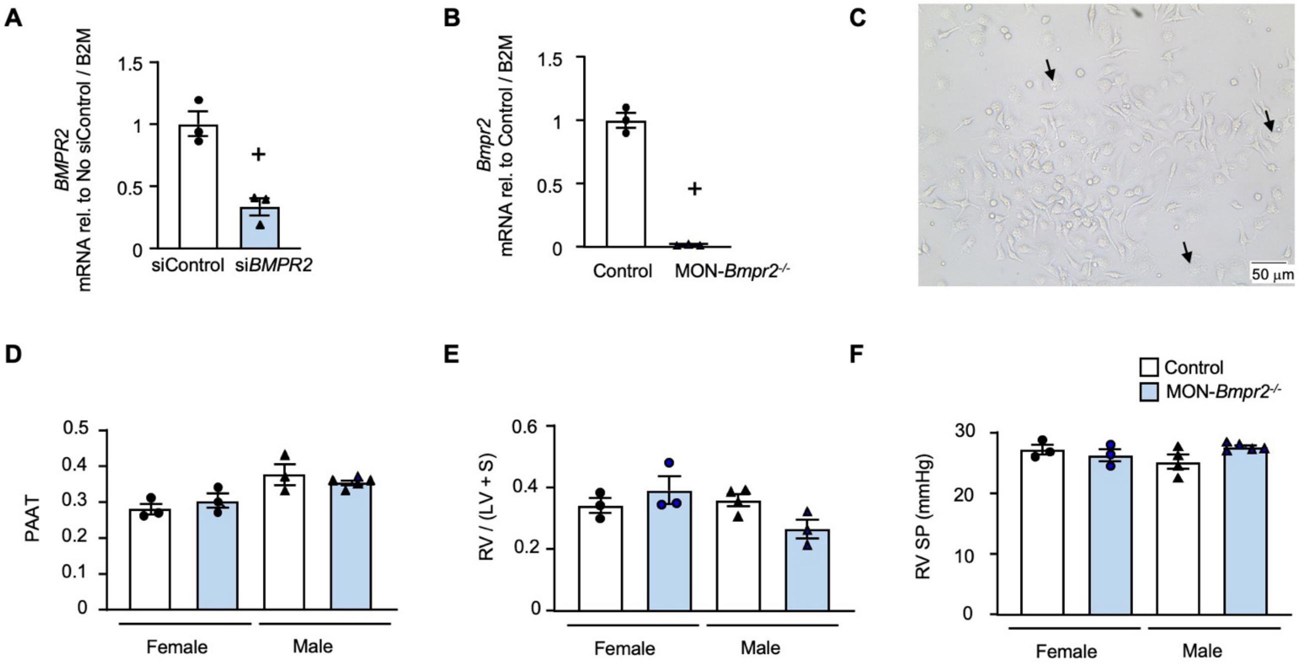


**Online Data Supplement Figure E4: Validation of BMPR2 knockdown and PAH assessments**

**(A)** Validation of *BMPR2* knockdown in THP1 following si*BMPR2* treatment. n=3 replicates. **(B)** Validation of *Bmpr2* decrease in circulating mouse monocytes following tamoxifen induced monocyte specific *Bmpr2* knockout in MON-*Bmpr2*$^{-/-/}$ mice compared to Control. **(C)** Representative image of mBMDM cells in culture with black arrow indicating granularity of macrophages. Induction of pulmonary hypertension is validated through: **(D)** Pulmonary artery acceleration time (PAAT) after hypoxia exposure. **(E)** Right ventricular hypertrophy (RVH) calculated as: right ventricle weight/ (left ventricle + septum weight) (RV/(LV+S). **(F)** Right ventricular systolic pressure (RVSP). Bars represent mean ± SEM. n=3 replicates in A, n=3 biological replicates in B and 3=5 mice per sex/ group in D, E and F with values after hypoxia exposure; + denotes the minimum achievable P-value for n=3 was reached by the nonparametric t test.

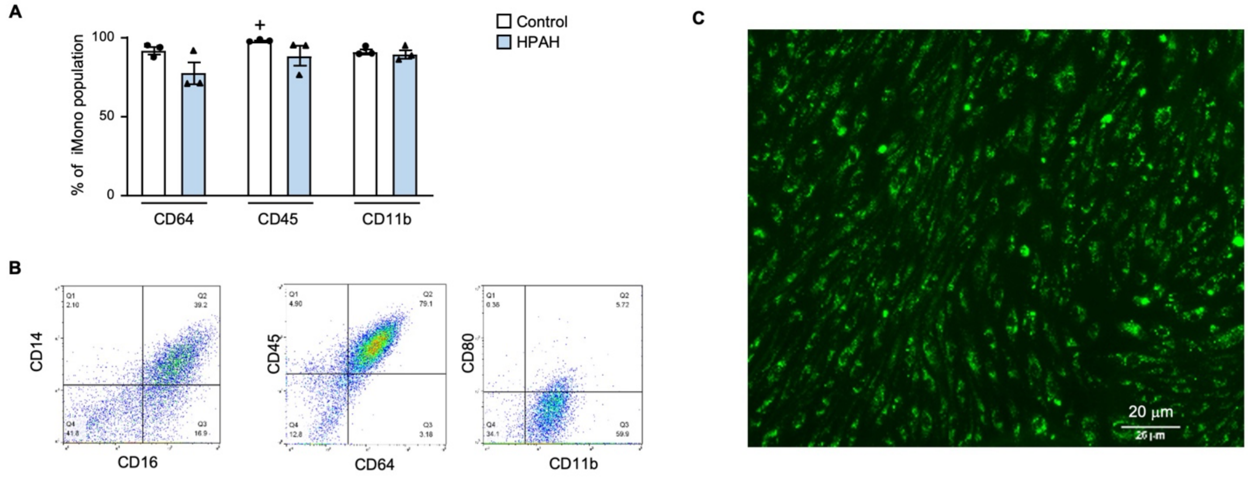


**Figure E5: iMono and iEC validation**

**(A)** FACS analysis of monocyte markers: CD64, CD45 and CD11b used to validate iMono. Bars represent mean ± SEM. n=3 biological replicates; + denotes the minimum achievable P-value for n=3 was reached by the nonparametric t test. **(B)** Gating strategy for iMono validation. **(C)** Representative iEC LDL uptake (green) image. Scale bar= 25 μm.
